# The Impact of Ellagitannins and Their Metabolites through Gut Microbiome on the Gut Health and Brain Wellness within the Gut–Brain Axis

**DOI:** 10.3390/foods12020270

**Published:** 2023-01-06

**Authors:** Roxana Banc, Marius Emil Rusu, Lorena Filip, Daniela-Saveta Popa

**Affiliations:** 1 Department of Bromatology, Hygiene, Nutrition, Faculty of Pharmacy, “Iuliu Hatieganu” University of Medicine and Pharmacy, 400349 Cluj-Napoca, Romania; 2 Department of Pharmaceutical Technology and Biopharmaceutics, Faculty of Pharmacy, “Iuliu Hatieganu” University of Medicine and Pharmacy, 400010 Cluj-Napoca, Romania; 3 Department of Toxicology, Faculty of Pharmacy, “Iuliu Hatieganu” University of Medicine and Pharmacy, 400349 Cluj-Napoca, Romania

**Keywords:** ellagitannins, ellagic acid, urolithins, ET-rich foods, gut–brain axis, antioxidant effects, anti-inflammatory effects, neuroprotective effects, anti-cancer effects

## Abstract

Ellagitannins (ETs) are a large group of bioactive compounds found in plant-source foods, such as pomegranates, berries, and nuts. The consumption of ETs has often been associated with positive effects on many pathologies, including cardiovascular diseases, neurodegenerative syndromes, and cancer. Although multiple biological activities (antioxidant, anti-inflammatory, chemopreventive) have been discussed for ETs, their limited bioavailability prevents reaching significant concentrations in systemic circulation. Instead, urolithins, ET gut microbiota-derived metabolites, are better absorbed and could be the bioactive molecules responsible for the antioxidant and anti-inflammatory activities or anti-tumor cell progression. In this review, we examined the dietary sources, metabolism, and bioavailability of ETs, and analyzed the last recent findings on ETs, ellagic acid, and urolithins, their intestinal and brain activities, the potential mechanisms of action, and the connection between the ET microbiota metabolism and the consequences detected on the gut–brain axis. The current in vitro, in vivo, and clinical studies indicate that ET-rich foods, individual gut microbiomes, or urolithin types could modulate signaling pathways and promote beneficial health effects. A better understanding of the role of these metabolites in disease pathogenesis may assist in the prevention or treatment of pathologies targeting the gut–brain axis.

## 1. Introduction

Numerous beneficial effects on human health, such as antioxidant, anti-inflammatory, anti-carcinogenic, cardioprotective, and prebiotic properties, have been reported following the consumption of many fruits, fruit juices, nuts, seeds, and beverages, these effects being due to their high content of antioxidant polyphenols, including tannins [1,2,3,4,5,6,7,8].

Tannins are secondary plant metabolites present in plants, foods, and beverages [9,10,11]. Among the parts of the plants that contain tannins are the bark, stems, roots, seeds, buds, leaves, and fruits [9,12]. Among foods containing tannins are fruits (grapes, blackberries, raspberries, strawberries, blueberries, cranberries, black and red currants, pomegranate, mangoes, apples, peaches, apricots, pears, guava) [9,13,14], juices [14,15], nuts (walnuts, cashews, hazelnuts, almonds, pecans, pistachios) [9,13,14], legumes (beans, lentils, cowpeas, peanuts, peas) [14,16], cereal grains (barley, rice, buckwheat, sorghum) [14], and beverages (wine [17], cognac [18], tea [19]).

Of the two main groups of tannins, namely, hydrolysable tannins and condensed tannins, ellagitannins (ETs) belong to hydrolysable tannins. They are non-flavonoid oligomeric polyphenols, which provide very high free-radical scavenging in vitro activity. While condensed tannins are composed only of flavonoids (flavan 3-ol or flavan 3, 4-diol), without a sugar core, hydrolysable tannins are composed of ellagic acid (EA) and gallic acid with a sugar core [9]. After hydrolysis, ETs release EA and both groups are associated with important health effects [20,21,22].

Taking into account the poor absorption of ETs and EA, and consequently, the negligible bioavailability of ETs, respectively, the very low bioavailability of EA, the beneficial effects observed following the consumption of ET-containing foods are explained by their extensive metabolism to urolithins. The urolithins circulate in the bloodstream and reach the systemic tissues at relevant levels [20,23]. Therefore, urolithins might be responsible for the health effects [24] attributed to the consumption of ET-rich foods, herbal teas (green and black *Camellia sinensis* teas), and herbal medicinal products [20,23,25].

Although the widespread presence of ETs in nature has been reported [26,27,28,29,30,31,32], consumer exposure to dietary ETs is relatively low, especially in some European countries, where the daily intake of ETs has been estimated to be around 5 mg/day, with berries being the main ETs contributors [33]. This is due to the relatively low ETs content in many food sources. In contrast, a higher daily ETs intake of 12 ± 37 mg/day was reported in the Finnish diet, with the largest dietary contribution to ETs intake in the Finnish cohort being strawberries [33]. Similar results were reported for the American diet, where the estimated dietary ETs intake was 12 ± 13 mg/day, with fresh berries contributing almost half of the estimated dietary ET intake (42%), while nuts, fruit juices, and preserves accounted for the other half of dietary ET intake [34]. Therefore, the consumption of ET-rich foods, such as berries and nuts, belonging to the group of functional foods, could have a positive impact on preventing lifestyle-related diseases.

ETs are food compounds that have been rather neglected by food scientists, nutritionists, and consumers until recently [13,35]. In the past, tannins were considered antinutritional compounds and were removed by food-processing techniques; nowadays, tannins are of interest due to their numerous biological activities [35,36].

The key property underlying the prevention and/or reduction of oxidative stress-related chronic diseases and the most discussed characteristic of polyphenols is their ability to scavenge reactive oxygen species (ROS), as well as oxidatively generated free radicals derived from important cellular macromolecules, such as lipids, DNA, and proteins [5,11]. Oxidative stress is due to an imbalance between the production and accumulation of ROS in cells and tissues and the antioxidant defense capacity, being the main mechanism responsible for several human diseases [5,37]. Unlike single-target drugs that alleviate symptoms without eradicating the cause, natural polyphenols from food sources have proven multi-target abilities to counteract oxidative stress [37]. In the case of ETs, the antioxidant efficiency is explained by the presence of several hydroxy functions in the ortho position and the strong ability to donate a hydrogen atom and support the unpaired electron [33]. Thus, ETs and EA demonstrated in vitro great potential in the treatment of oxidative stress-mediated human diseases, comparable to that of urolithins, but in vivo-only urolithins proved to be responsible for the beneficial effects [37].

As is well known, oxidative stress and inflammation are closely related pathophysiological processes, each of them being able to easily induce the other, and both occur simultaneously in many pathological conditions, including diabetes and diabetic complications, hypertension and cardiovascular diseases, neurodegenerative diseases, alcoholic liver diseases, chronic kidney diseases, cancer, and aging [38,39]. In addition to their antioxidant effects, ETs and EA have shown strong anti-inflammatory activities [3,21,40,41]. Therefore, several studies have managed to confirm the effectiveness of ETs and EA in the treatment of chronic inflammatory diseases and conditions [42,43,44].

The scientific insights presented in this review about the in vivo metabolisms of ETs, but also about the antioxidant and anti-inflammatory, neuroprotective, and anti-cancer activities of ETs and their metabolites, tend to support the hypothesis that the potential health benefits of dietary ETs could reduce the impact of chronic and degenerative diseases and aging-related diseases, such as cancer, diabetes, cardiovascular diseases, and central nervous system disorders [45].

In the present review, we aimed to report relevant and recent aspects of the role of ETs and their microbiota-derived metabolites in gut and brain health documented on in vitro, in vivo, and in some new clinical studies.

We conducted a narrative review of the literature using the academic databases Pubmed and ScienceDirect for the search and collection of literature. Major keywords, such as “polyphenols“, “hydrolysable tannins“, “ellagitannins“, “ellagic acid“, “urolithins“, “chemical structure“, “sources“, “metabolism“, “bioavailability“, “biological activities“, “antioxidant“, “anti-inflammatory“, “neuroprotective”, “anti-cancer”, “gut”, “intestine”, “brain”, “gut–brain axis”, and “in vitro“, “in vivo“, and “clinical studies“ were used individually or in combination during the literature survey. We considered original research articles written in English and based our search on their importance and relevance to the field. As it was necessary to focus on the most impactful and relevant aspects, we included published review articles and book sections only where appropriate. We selected publications between January 2000 and October 2022 but generally focused on the most recently published articles. The non-English publications were excluded.

## 2. Chemistry of Ellagitannins

ETs are esters of hexahydroxydiphenic acid (HHDP) with polyols, such as glucose or quinic acid [46]. By hydrolysis of ETs, with acids or bases, HHDP is obtained, which is spontaneously lactonized to EA [22].

EA is a dilactone formed by combining two molecules of gallic acid [47]. Following glycosylation, methylation, or methoxylation of EA hydroxyl groups, numerous ETs derivatives appear in plants [33,46].

Depending on the number of HHDP groups present in the molecule, ETs can be classified into monomeric, oligomeric, and polymeric ETs [12,35]. Monomeric ETs are made up of a single HHDP group bound to a glucoside core [12]. Monomeric ETs tend to polymerize, forming dimers, oligomers, and polymers, in which the monomeric units are C-O-C bonded [12,48]. In addition, ETs are grouped into monomeric (e.g., nupharin A, geraniin, tellimagrandin II, punicalagin, eugeniin, davidiin, casuarictin, and corilagin), dimeric (e.g., sanguiin), oligomeric (e.g., agrimoniin, nupharin E, nupharin C, and hirtellin A), and C-glycosidic ellagitannins (e.g., vescalagin, castalagin, casuarinin, and stachyurin) [14,48].

**Figure 1:**
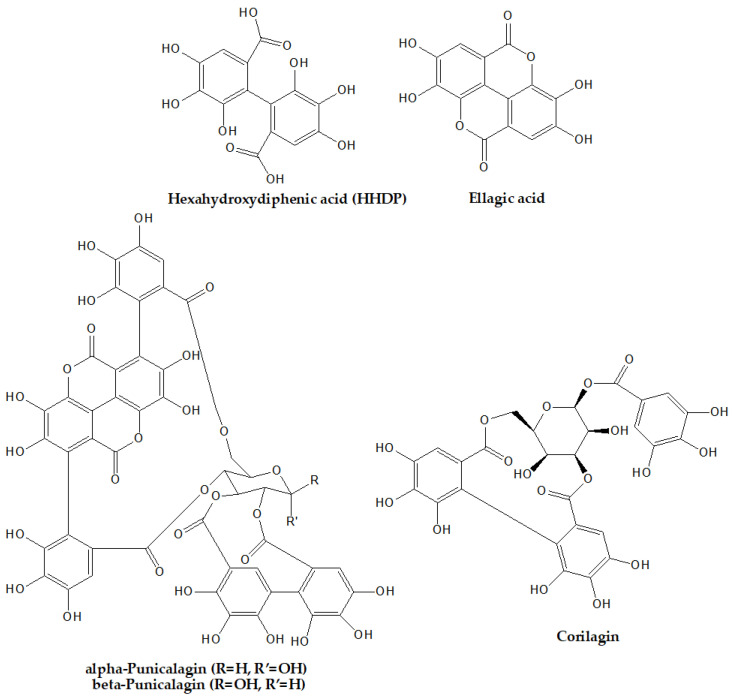
The chemical structures of some ellagitannins and ellagic acid.

There is a great structural diversity among ETs that depends on the variations in the position, frequency, and stereochemistry of the HHDP units, the galloylation degree, and/or the anomeric stereochemistry of the sugar moieties [33,35]. The high structural diversity and complexity of the ET molecules (Figure 1) influence their hydrolytic susceptibility, but also the ease with which ETs will undergo various chemical reactions, such as transformation, isomerization, and oligomerization [35,48]. The chemical structure of ETs has a great influence on their efficiency as antioxidants, the efficacy being related to the degree of hydroxylation and decreasing with the presence of a sugar moiety [33].

## 3. Sources of Ellagitannins

Among the hydrolysable tannins, ETs are found in more plant families compared to gallotannins [14,49]. Although over 1000 natural ETs have been identified in nature to date, only a few of them are predominant in foods [35,50]. As previously mentioned, the main food sources of ETs are fruits, nuts, and seeds, but ETs may also be present in herbs, roots (used as folk medicine), and alcoholic beverages matured in wooden barrels, while EA has also been found in some types of honey [14,35,48,51].

Among fruits, abundant sources of ETs are several berries, such as raspberries, blackberries, cloudberries, blueberries, cranberries, gooseberries, currants, and strawberries [48,51]. ETs are also found in many other fruits, including pomegranates, mangos, persimmons, guavas, plums, apricots, peaches, bird cherries, and muscadine grapes [35,46]. In addition, some fruits consumed in Brazil have been reported as food sources of ETs [52,53]. Of these, the highest ET content was reported for jabuticaba, grumixama, and cambuci, while Camu-camu (Amazonian fruit) and Surinam cherry (Brazilian cherry), respectively white and red guavas, showed intermediate and low levels of ETs [52]. Moreover, rambutan (*Nephelium lappaceum* L.) peels are a potential source of ETs [54].

Regarding alcoholic beverages, EA and ETs are the most representative phenolic compounds of wine and spirits aged in barrels. ETs can represent up to 10% of the dry weight of the oak heartwood, being transferred to the wine during aging [17,55,56]. All the eight ETs identified in the traditional oak species, namely castalagin, vescalagin, granidin, and roburins (A, B, C, D, and E), were found in wines aged in oak barrels, the first two being generally the most abundant and representing nearly 50% of the total ETs [17,18,55,57,58]. Once in the wine, the ETs undergo continuous transformations, such as reactions with the components of the wine, as well as oxidation and hydrolysis reactions, or may be involved in tannin condensation [17,55,58]. The products resulting from these reactions contribute to the feeling of bitterness and astringency and could affect the wine color [17,56,58]. In particular, ethyl derivatives, such as β-1-*O*-ethylvescalagin, complexes colored red-orange with anthocyanins that alter the wine color, and also flavonoids, such as flavano-elagitanins (e.g., acutissimin A, acutissimin B, epiacutissimin A, and epiacutissimin B) [17,55,58]. Wines aged in oak have high levels of hydroxybenzoic acid derivatives, in particular EA, which comes from the hydrolytic decomposition of oak ETs [46]. It has been reported that young cognac “eaux-de-vie” aged in barrels also contains all eight ETs mentioned above [18].

The occurrence of ETs and EA in some natural products is shown in Table 1.

## 4. Metabolism and Bioavailability of ETs and EA

Unlike condensed tannins, there are fewer studies on the absorption, metabolism, and bioavailability of ETs in humans. In contrast, animal studies have provided more information.

According to bioavailability studies, it appears that ETs are not absorbed as such due to their increased size and polarity [11,35,68]. ETs that are sensitive to acidic hydrolysis in the stomach and basic hydrolysis in the duodenum release the bislactone EA, which is poorly absorbed, while hydrolysis-resistant ETs end up almost intact in the large intestine [11,35,69].

Poor intestinal absorption of EA has been observed in some human studies, in which, after oral administration of pomegranate juice, low plasma concentrations of EA were detected and no intact forms of ETs. The likely explanation for the poor absorption of EA is its low water solubility and its ability to complex calcium and magnesium ions in the gut [14,35,70].

In the lower gastrointestinal tract, released EA is converted to dimethylated ellagic acid glucuronide, which is further metabolized by the human intestinal microbiota (*Gordonibacter urolithinfaciens*, *Gordonibacter pamelaeae,* and *Ellagibacter isourolithinifaciens*) to hydroxy derivatives of dibenzopyran-6H-6-one, called urolithins [11,35,48,71,72].

**Figure 2:**
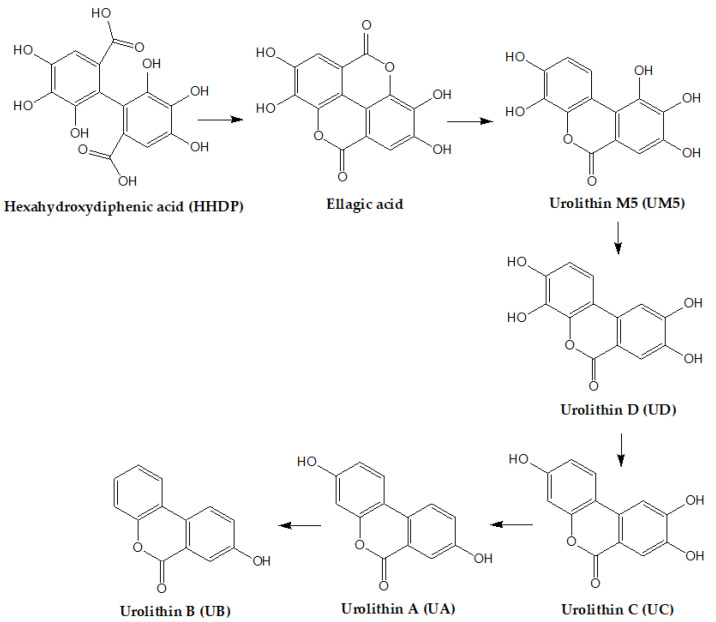
Ellagic acid and some of its microbial metabolites, the urolithins.

Gut microbiota can convert EA into urolithins (Figure 2) via lactone ring cleavage, decarboxylation, and dehydroxylation reactions, starting with the metabolite pentahydroxy-urolithin and continuing with tetrahydroxy- (urolithin D (UD), urolithin E (UE) and urolithin M6 (UM6)), trihydroxy- (urolithin C (UC) and urolithin M7 (UM7)), dihydroxy- (urolithin A (UA) and iso urolithin A (iso-UA)), and monohydroxy- (urolithin B (UB)) dibenzopyran-6-one metabolites [25,51,73]. The urolithins are further incorporated into the enterohepatic circulation [48].

Following the consumption of ET-rich foods, the urolithins were found in human plasma and urine [14,35]. Since studies have shown a long-term persistence of urolithins in the body after dietary intake of ETs, the urolithins are considered bioactive forms of ETs and EA, responsible for the anti-cancer effects demonstrated by food ETs in vivo [11,35,69].

However, the inter-individual variability of the colonic microbiota affects the metabolism and bioavailability of ETs, leading to inter-individual variations in urolithins production [14,35,69,74]. Thus, in studies performed on healthy volunteers, “low excreters” and “high excreters” of urolithins were identified, respectively [33,35]. Recently, a stratification of individuals according to their urinary urolithin excretion status has been proposed [23,25,35]. Thus, three gut microbiota-associated metabotypes were defined: metabotype A, which includes those producers of only UA as the final urolithin; metabotype B, which includes subjects who produce UB and iso-UA in addition to UA; and metabotype 0 (corresponding to “low excreters”), individuals that cannot produce final urolithins, in their case only the pentahydroxy-urolithin precursor is detected [21,23,25].

While metabotype 0 was reported to be approximately constant (10%) in a large age range (5-90 years), for the other two metabotypes the distribution is significantly influenced by age, as follows: metabotype A being predominant at early ages (85%) and decreasing in adulthood (up to 55%), with the parallel increase in metabotype B (from 15% to 45%). After the age of 40, the proportion of the three metabotypes (0, A, and B) remains unchanged (10%, 55%, and 45%, respectively) [25,75].

In addition to age, the main determining factor of the distribution of urolithin metabotypes in the population, other factors, including diet, physical activity, health status, sex, weight, or body mass index, could have a potential influence.

Regarding the diet, it has been observed that it can modulate to a certain extent the gut microbiota involved in the ETs-EA metabolism pathway. Thus, following a chronic consumption of pomegranate extract with a high content of ETs (1.8 g extract containing 425 mg ETs), non-producer individuals (metabotype 0) became producers (metabotype A or metabotype B) [76].

Cortés-Martin et al. [77] also reported a significant association between increased physical activity and the prevalence of metabotype B, especially between 5 and 18 years. In contrast, these authors observed no relationship between metabotype and sex.

The association between metabotype and disease risks was revealed in a study that included only adults. In their case, the prevalence of metabotype B increased in people with chronic illness (metabolic syndrome or colorectal cancer) associated with gut microbial imbalance (dysbiosis) [78]. When larger age ranges were considered, the association was no longer confirmed [77].

Another study described a potential correlation between the prevalence of metabotype B and obesity. Thus, the gut dysbiosis associated with overweight and obesity affected the metabolism of EA and ETs, the percentage of metabotype B individuals, characterized mainly by the production of iso-UA and/or UB from EA, being more abundant in overweight-obese than in normal-weight subjects [79].

These differences observed in the distribution of urolithin metabotypes may be the result of differences in the human gut microbiome associated with health status.

Despite the potential associations that could explain the differential capacity of individuals to metabolize EA derivatives into urolithins, some authors argue that the distribution of urolithin metabotypes in the population is mainly determined by aging, which indicates that the gut microbiota involved in the metabolism of ETs and EA is developmentally regulated [77,80].

Consequently, the biological activities of ET-rich foods, such as anti-inflammatory and anti-cancer effects, may differ from person to person, depending on the composition of the intestinal microbiota [74].

## 5. Biological Activities and Mechanisms of Action of ETs, EA, and Urolithins

The mechanisms of action underlying the biological activities of ETs are not fully elucidated even at present. The hypothesis first launched by Cerdá et al. [81] in 2005 is still supported. According to it, urolithins and/or their possible phase II conjugates formed in vivo are responsible for the biological activities of dietary ETs. Their anti-cancer, anti-inflammatory, cardio-metabolic, antioxidant, and neuroprotective effects [20,25,81] have been demonstrated following in vitro testing, but the in vivo studies are still limited [25,45].

### 5.1. Antioxidant Activity of ETs, EA, and Urolithins

Numerous health benefits reported for EA, including anti-inflammatory, cardioprotective, neuroprotective, hepatoprotective, gastroprotective, nephroprotective, antibacterial and antiviral properties, cancer preventive and suppressive effects, respectively inhibition of oxidative stress, were partially attributed to the antioxidant activity of EA [37,82,83,84]. EA is considered a very effective antioxidant, with a similar scavenging activity to other major antioxidants, such as vitamins C and E [39,82,84]. Due to its ability to regenerate and not being reduced after metabolism, EA can provide continuous protection against oxidative stress, even at micromolar concentrations [84].

Taking into account its modes of action, EA can be considered an antioxidant with multiple functions [37].

Thus, EA can act as a primary antioxidant (type I, or chain breaking), i.e., acting as a free-radical scavenger, based on its ability to transfer the phenolic H atom to a free radical, exerting its antioxidant effects mainly through three mechanisms of action, namely: single electron transfer (SET), hydrogen atom transfer (HAT) and sequential proton loss hydrogen atom transfer (SPLHAT), respectively [37,84,85,86].

Also, EA can act as a secondary antioxidant (type II, or preventive), exerting its effects against free radicals due to its ability to inhibit the endogenous production of oxidants and especially of the hydroxyl radical (•OH), the most reactive and electrophilic among oxygen-based radicals [37].

The secondary antioxidant activity of EA is also related to EA’s ability to chelate ionic metals such as copper, iron, nickel, and cadmium, providing an additional mechanism of protection against oxidative stress [37,82]. Thus, by chelating and subtracting metals such as Fe^2+^, Fe^3+^, and copper ions involved in the production of free radicals, EA prevents low-density lipoprotein (LDL) oxidation [37,84].

In addition to scavenging prooxidant agents, EA can modulate many cell signaling pathways. It has the ability to activate the nuclear factor erythroid 2-related factor 2/antioxidant response element (Nrf2/ARE) pathway that has a major role in cytoprotection [87]. Thus, EA increases the activity of some antioxidant and detoxifying enzymatic systems (superoxide dismutase (SOD), glutathione peroxidase (GPx), glutathione reductase, and catalase (CAT)) by regulating the Nrf2 redox-sensitive transcription factor, after UV-B light-induced oxidative stress in human dermal fibroblast [88,89].

Its structure, with two lactones and four phenolic hydroxyl groups that have the ability to produce hydrogen-bonding interactions, allows it to inhibit also some enzymes involved in the radical generation, such as various cytochrome P450 isoforms, lipoxygenases, cyclooxygenase (COX) and xanthine oxidase, thereby inhibiting the excessive production of ROS and reactive nitrogen species (RNS) [39,82,90,91].

However, through some of its hydroxyl groups, EA can exert prooxidant activity under certain conditions (such as high doses, high concentrations of transition metal ions, alkaline pH, or the presence of oxygen molecules) [37,92,93,94]. This is specific to small polyphenols and appears little or not at all in the case of high molecular weight polyphenols, such as ETs [94].

The most used methodologies reported evaluating the chemical antioxidant capacity of the phenolic compound family, including ETs and their metabolites, are the 2,2′-azinobis-(3-ethylbenzothiazoline-6-sulfonic acid) (ABTS), 2,2-diphenyl-1-picrylhydrazyl (DPPH), the ferric reducing ability of plasma (FRAP) and oxygen radical absorbance capacity (ORAC) assays [21]. Of these, the ABTS, DPPH, and FRAP assays are based on the SET mechanism, while the ORAC assay measures antioxidant inhibition of peroxyl radical-induced oxidation, thus reflecting classical radical chain-breaking antioxidant activity by the HAT mechanism [95].

Most studies point to a strong correlation between antioxidant properties and the structure of ETs [14]. Thus, the antioxidant and free-radical scavenging capacity increases as the degree of polymerization increases, being stronger for high molecular weight ETs [14,96]. This was explained by the presence of several hydroxy functions in the ortho, which have a greater ability to donate a hydrogen atom and support the unpaired electron, in the case of high molecular weight ETs, compared to those with low molecular weight [33,97].

It is considered that among the hydroxy groups present in the structure of high molecular weight ETs, especially the multiple pyrogallol-type galloyl units have a major contribution to the particularly strong antioxidant activity [11,96]. Therefore, Pfundstein et al. [98] reported a stronger DPPH scavenging capacity and FRAP for punicalagin and punicallin, which have a higher degree of hydroxylation than EA. Similar results were obtained by Seeram et al. [97] in the Trolox equivalent antioxidant capacity (TEAC) assay, namely, a higher antioxidant capacity for punicalagin compared to EA.

On the other hand, the results obtained by Sun et al. [99] express the opposite for the DPPH scavenging test and the FRAP test, where the trends for activities decreased in the order of EA > punicalagin > punicallin and only when acting against O_2_^−^, punicalagin and punicallin showed much higher activities than EA, and their relative activities decreased in the order of punicalagin > punicallin > EA. Despite the observed differences, the results confirmed the strong antioxidant capacities of the three compounds, punicalagin, punicallin, and EA.

Comparing punicalagin, punicallin, and corilagin in the ORAC, FRAP, and DPPH assays, Pfundstein et al. [98] found that punicalagin, with a total of 16 gallic hydroxyl groups, was the best in the ORAC and FRAP assays, corilagin with only 9 hydroxyls was the best in the DPPH assay, while punicallin with 10 hydroxyls in the highly restrained gallagyl unit was the weakest in all 3 assays, molecular flexibility proving to be the key factor and the decrease in the inhibitory activity taking place with the increase in the rigidity of the molecule.

Regarding the presence of free galloyl groups in the structure of ETs, it was observed that the radical-reducing power was stronger in the case of ETs with a higher number of free galloyl groups; thus, the HO∙ scavenging capacity of telimagrandin II, which has three galloyl groups, was better than that of telimagrandin I, with only two galloyl groups [96]. In contrast, dimeric ETs agrimoniin and gemin A demonstrated similar scavenging activities, despite the different number of galloyl groups [96]. These different results may be due to different molecular characteristics of the compounds, such as spatial structure and solubility, but also to different radical properties [96,99].

As an example, Gulcin [100] reported that low molecular weight compounds more easily access the radical site, being more active in scavenging DPPH, while high molecular weight compounds may react slowly or even be inert in the DPPH scavenging capacity.

Even though some ETs have shown strong antioxidant effects in vitro, they cannot be extrapolated in vivo due to the metabolism of ETs and EA by the intestinal microbiota to urolithins, with an antioxidant activity different from that of ETs.

This was confirmed in the study by Sun et al. [99], where oxidative stress was induced in mice with oxidized fish oil. Thus, while administration of punicallin and punicalagin resulted in significant reduction of oxidative stress (decreasing the malondialdehyde (MDA) level, increasing the activities of GPx and SOD enzymes) only in the gut, EA had the most effective protective effects against oxidized fish oil-induced oxidative damage in all tested tissues, namely intestine, plasma and liver. Therefore, punicalagin and punicallin are not absorbed into the bloodstream due to their large size and cannot exert their full antioxidant potential in other tissues than the intestine where they are hydrolyzed to EA and metabolized to urolithins by the intestinal microbiota [99,101,102].

Panchal and Brown [102] showed that an extract from European oak bark used in red wine maturation containing a mixture of ETs (vescalagin, castalagin, roburin E, grandinin, and EA) improved oxidative stress markers in high-fat diet-fed Wistar rats. Thus, reduced plasma concentrations of MDA and increased plasma GPx activity revealed a marked antioxidant response and protective effects mediated by oak-derived ETs, similar to those produced by pomegranate-derived ETs.

Considering the strong antioxidant properties demonstrated by foods rich in ETs, exploring the antioxidant capacity of urolithins has become a research topic of great interest in recent years. The urolithins most commonly found in humans and animals are UA, iso-UA, and UB [37].

Compared to ETs, urolithins demonstrated a modest in vitro antioxidant activity, with variable activity values, depending on the tested metabolite and the assay used [37,103,104].

Thus, the first study supporting these claims, conducted by Cerda et al. [103], reported an antioxidant activity of the metabolite 3,8-dihydroxy-6H-dibenzo[*b,d*]pyran-6-one (UA) of 42-fold and 3570-fold lower than that of punicalagin with DPPH and ABTS assays, respectively. Recent studies also reported for UA an antioxidant activity 23-fold lower than that of EA, in the DPPH assay [104,105].

The results obtained by other antioxidant tests, such as FRAP or thiobarbituric acid reactive substances (TBARS), did not confirm the antioxidant activity of UA in plasma or colon mucosa [15].

However, using the ORAC assay, Ito [106] reported that all tested urolithins exhibited strong antioxidant properties compared to ascorbic acid, with UA being the strongest among them. In addition, metabolites exhibited more potent antioxidant activities in the ORAC assay than in intact ETs, such as geraniin and corilagin. In addition, following oral administration of geraniin to rats, plasma ORAC scores increased with increasing plasma UA concentration.

In another similar study, comparing the antioxidant activities of polyphenol metabolites with those of intact functional polyphenols geraniin, chlorogenic acid, and (−)-epigallocatechin gallate (EGCG) by an ORAC assay, stronger antioxidant activity was reported for the metabolites than for their original compounds, with UA showing the most potent antioxidant activity among all metabolites [107].

The greater antioxidant capacity of urolithins compared to EA was also confirmed in the study carried out by Kallio et al. [95], where the ORAC values for EA and urolithins A and B were 4.25, 6.67, and 5.77 Trolox equivalents, respectively, and the ORAC_UA_/ORAC_EA_ ratio was 1.57.

Thus, the antioxidant capacity of urolithins proved to be questionable following the results obtained through the DPPH, FRAP, and ABTS antioxidant assays (all based on the SET mechanism), while only the ORAC assay identified urolithins as antioxidants [37,95,104]. Unlike EA, which, due to its chemical structure, can exert its antioxidant effects both through SET and HAT reactions, unconjugated urolithins, having no other functional groups in their structure than one (UB) or at most two phenolic hydroxyl groups (UA), that act as hydrogen donors, will exert their antioxidant effects only through HAT reactions [82,84,95]. Since the electron-withdrawing carboxyl group of urolithins is part of a lactone ring, it cannot promote the SET mechanism [37,95].

Just based on the fact that they exert their antioxidant effects exclusively by the HAT mechanism, urolithins cannot be considered less potent antioxidants, especially since the HAT mechanism and oxidation induced by peroxyl radicals are considered to be more biologically relevant than the SET mechanism or oxidation induced by other oxygen radicals [95,108].

Therefore, the assumption that urolithins could have a higher antioxidant capacity than originally thought is still being investigated, aiming to prove that these metabolites can have an important contribution as antioxidants in the body after the oral administration of intact ETs.

### 5.2. In Vitro Studies Attesting to the Beneficial Influence of ETs and Their Metabolites on the Gut–Brain Axis

ETs, high molecular weight polyphenols, are metabolized by animal and human gut microbiota to bioavailable urolithins, low molecular weight metabolites. As urolithins rapidly undergo phase II metabolism following gut absorption, the in vitro studies should be conducted on urolithins, as well as their phase II respective glucuronides to fully evaluate the biological activity mechanisms.

The gut–brain axis is a bidirectional communication system involving many signaling molecules between the enteric and central nervous system (CNS). Scientific evidence suggests that crosstalk along the gut–brain axis regulates inflammatory responses, antioxidant activity, cell growth, and proliferation, modulating the homeostasis of functions in these systems. Alterations in gut–brain interactions have been recognized in several digestive and neurological disorders [109]. However, psychobiotics, bacterially mediated molecules including prebiotics, probiotics, and postbiotics, have the potential to affect microbiota–gut–brain axis signaling [110]. ETs and their postbiotic metabolites are part of the dietary intervention factors that can influence the gut–brain axis and be part of the prevention and therapeutic approaches [111].

#### 5.2.1. Antioxidant and Anti-Inflammatory Effects

Oxidative phosphorylation (OXPHOS), a metabolic pathway that produces energy inside mitochondria, could generate an over-production of ROS under stressful conditions revealing a link between mitochondrial function and aging [112]. If the antioxidant homeostasis systems do not work properly, ROS could accumulate and induce oxidative stress and inflammatory processes, lipid peroxidation in cell membranes, initiation of mitochondrial dysfunctions, and cell apoptosis, all essential risk factors involved in the pathogenesis of many chronic diseases, including neurodegenerative diseases, cardiovascular disease, or cancer [113]. Antioxidant and anti-inflammatory pathways are basically intertwined, affecting similar biomarkers. Increased ROS concentration triggers proinflammatory signaling and mediators, such as nuclear factor kappa B (NF-κB) and COX-2, that generate inflammatory cytokines, including interleukins IL-1β, IL-6, IL-8, and tumor necrosis factor α (TNF-α). Nevertheless, ETs and their metabolites could induce antioxidant and anti-inflammatory responses through scavenging free radicals and ROS [45]. Thus, these compounds were shown to silence the NF-κB and mitogen-activated protein kinase (MAPK) proinflammatory signaling pathways or upregulate heme oxygenase-1 (HO-1) expression by activation of the phosphatidylinositol 3-kinase (PI3K)/protein kinase B (Akt) and Nrf2/ARE pathways [114].

In SK-N-MC human neuroblastoma cell line, UA significantly attenuated intracellular ROS production and oxidative stress-induced apoptosis, increased cell viability, decreased the Bax/Bcl-2 ratio, and suppressed the p38 MAPK pathway phosphorylation [115]. Additionally, UA protected mitochondrial function and alleviated oxidative stress via the SIRT1/PGC-1α pathway [116]. A new study confirmed that UA could inhibit ROS generation, increase the levels of Nrf2, manganese superoxide dismutase (MnSOD), and total glutathione (GSH), and reduce TNF-α, NF-κB, and IL-6 levels by activating SIRT1 signaling [117].

Another UA anti-inflammatory activity mechanism was mediated through the aryl hydrocarbon receptor (AhR), a ligand-activated transcription factor implicated in numerous physiological and pathological cellular mechanisms, such as immunity, energy homeostasis, and epithelial barrier function [118]. The study of Singh et al. [119] showed that UA significantly enhanced gut barrier function and inhibited inflammation via activation of AhR-Nrf2-dependent pathways that increased epithelial tight junction proteins. It was exposed that gut-derived metabolites presented anti-inflammatory AhR ligand activities and, through targeting the gut–brain axis, could treat neuroinflammatory diseases [120]. These AhR agonists generated by the gut microbiome can cross the blood-brain barrier and diminish CNS inflammation via activating AhR ligands [121]. Recent evidence showed that urolithins, especially UA, could play key roles in multiple sclerosis (MS) pathogenesis through alteration of the intestinal epithelial barrier function [122]. These results were confirmed by Hering et al. [123], revealing that UA could reverse the proinflammatory dysregulation induced by TNF-α in colonic HT-29/B6 cells, while in ileum-like Caco-2 cells, EA strengthened barrier function, a key feature of intestinal health. Besides reinforcing the barrier function per se, Iglesias et al. [124] showed that EA could also inhibit TNF-α triggered negative effects, including IL-6 and IL-8 release, increased intercellular adhesion molecule-1 (ICAM-1) and Nod-like receptor protein 3 (NLRP3) expression, or increased mitochondrial oxidant production. Mechanistically, EA acted primarily through the inhibition of NF-κB and extracellular signal-regulated kinases (ERK) 1/2 pathways, breaking the cycle of inflammation and oxidative stress. This is in agreement with previous in vitro results in which UA significantly decreased the expression of proinflammatory cytokines IL-6 and TNF-α through modulation of miR-27 expression and the ERK/peroxisome proliferator-activated receptor gamma (PPAR-γ) signaling pathway [125].

Knowing that endothelial Akt-kinase plays an important role in the pathogenesis of cardiovascular complications in diabetic patients, the effects of different polyphenols, including UA, on Akt phosphorylation (pAkt) in endothelial cells were assayed [126]. UA, but not UB, UC, or UD, was among the strongest pAkt inhibitors, which can be linked to the two OH-groups at the C3 and C8 positions in the UA structure. Moreover, UA may even be considered a therapeutic candidate against diabetic podocytopathy, as UA treatment enhances podocyte viability and reduces ROS levels [127].

The in vitro anti-inflammatory activities of UA, iso-UA, UB as well as their respective glucuronides were performed on THP-1 human cell line-derived macrophages, RAW 264.7 murine macrophages, and human primary macrophages. UA, the most active metabolite, inhibited the human immune cell inflammatory response induced by TLR4 receptor stimulation and increased the production of anti-inflammatory cytokines, IL-10 and transforming growth factor beta 1 (TGF-β1). The anti-inflammatory activity mechanism was based on the inhibition of NF-κB translocation to the nucleus and MAPK phosphorylation contribution [128].

Further studies on RAW 264.7 cells showed that UA treatment prevented NF-κB and AP-1 activation, inhibited Akt and Jun N-terminus kinase (JNK) phosphorylation, significantly diminished the intracellular accumulation of ROS, reduced the activation of NADPH oxidase (NOX), the main source of ROS in activated macrophages [129]. These outcomes confirmed that, in lipopolysaharide (LPS)-challenged RAW 264.7 murine macrophages, UA could lower nitric oxide (NO) production through inhibition of the induced NO synthase (iNOS) protein, decreased the expression of TNF-α, IL-1β, and IL-6 mRNA, besides inhibiting NF-κB p65 nuclear translocation and p50 DNA-binding action, processes associated with anti-inflammatory activity [130,131].

In murine J774.1 macrophages, UA also inhibited the production of proinflammatory proteins, ROS, and NO, and blocked p65 NF-κB nuclear translocation [132].

A common anti-inflammatory strategy includes the decrease in biosynthesis of prostaglandins and leukotrienes via inhibition of the COX-2 and 5-lipoxygenase (5-LOX) pathways. A recent in vitro study revealed that UA, iso-UA, and UC could reduce the formation of the 5-LOX/COX-2 pathway hemiketal eicosanoids (HKE_2_ and HKD_2_), novel mediators of inflammation [133].

UA might be used as a natural immune-suppressant in some inflammatory diseases, as UA treatment decreased CD4^+^ T cell proliferation and upregulated the miR-10a-5p expression, which in turn restrained store operated Ca^2+^ entry (SOCE) and suppressed the activation of murine CD4^+^ T cells [134].

#### 5.2.2. Neuroprotective Effects

There are many in vitro studies that assessed the neuroprotective effects of EA or urolithins an different line cells in different experimental conditions and revealed several molecular mechanisms of action.

Investigations on LPS-stimulated BV2 microglia cells confirmed that UA significantly reduced the production of proinflammatory cytokines, TNF-α and IL-6, via the activation of SIRT-1 and autophagy initiation [135]. Equally, UA treatment attenuated neuroinflammation in BV-2 microglia, decreased proinflammatory cytokine expression, reduced inducible nitric oxide synthase gene expression, and suppressed JNK/c-Jun pathway activation [136]. Moreover, UB inhibited the TNF-α, IL-6, and IL-1β concentrations and suppressed NF-κB activity and phosphorylation of JNK, ERK, and Akt. In addition, UB increased the production of anti-inflammatory cytokine IL-10 and the phosphorylation of AMP-activated protein kinase (AMPK), associated with anti-inflammatory and antioxidant activities [137].

It was suggested that the potential mechanisms of the anti-inflammatory activity of UA and UB in LPS-treated BV2 murine microglia could be via the inhibition of NF-κB p65, Erk1/2, p38 MAPK, and Akt phosphorylation and signaling pathways. The two urolithins suppressed mRNA levels of proinflammatory TNF-α, IL-6, IL-1β, iNOS, and COX-2 genes [138].

Interestingly, in an experimental autoimmune encephalomyelitis (EAE) study, the most common model for MS, the EA treatment did not affect the cause of inflammation like urolithins did but rather the consequences, such as demyelination, through the stimulation of ceramide biosynthesis [139].

The characteristic features of the Alzheimer’s disease (AD) brain are the accumulation of amyloid beta (Aβ) in extracellular senile plaques and intracellular hyper-phosphorylated tau protein, as well as oxidative stress accompanied by mitochondrial dysfunction [140].

As mitophagy deficit is a key factor in AD mitochondrial dysfunction, the use of urolithins can restore mitochondrial homeostasis by inducing mitophagy and biogenesis [24]. Activation of SIRT, AMPK or PGC1-α pathways and inhibition of mammalian target of rapamycin (mTOR) modulated by UA treatment-induced mitophagy and mitochondrial biogenesis [141].

One of the main strategies for the treatment of AD is the inhibition of Aβ-induced neurotoxicity. An in vitro study revealed that EA and its metabolites, UA, UB, UM5, UM6, attenuated the Aβ-induced toxicity in PC12 cells promoting neurite outgrowth, reducing ROS production, and inhibiting neuronal apoptosis. The results identified AKT1, Insulinlike growth factor1 receptor (IGF1R), NFKB1, epidermal growth factor receptor (EGFR), and MAPK14 genes as main targets of EA and urolithins, therefore, the possible neuroprotective action mechanisms were associated with PI3K-Akt, MAPK, and Ras signaling pathways [142].

Another approach for the prevention of AD is the regulation of mitochondrial calcium influx and mitochondrial ROS (mtROS) accumulation, which were noticed in diabetic patients. In neuroblastoma cell line SH-SY5Y, UA treatment reduced high glucose-induced amyloidogenesis, maintaining mitochondrial calcium and ROS homeostasis [143]. Additionally, UA treatment increased expression of genes of mitochondrial biogenesis and OXPHOS, suggesting hormetic effects [144].

UB presented suitable radical scavenging potential against ABTS, DPPH, OH^−^ and O_2_^−^, thus could modulate oxidative stress, a significant factor in the progression of chronic diseases, such as dementia [145]. Moreover, on H_2_O_2_-treated neuron-2a cells, UB significantly reduced apoptosis rate and ROS production, and increased viability.

Monoamine oxidase (MAO) enzymes (MAO-A and MAO-B) are responsible for the inactivation of monoamine neurotransmitters and could generate neurological disorders, such as depression and Parkinson’s disease (PD). Treatment with UA, UB and UC significantly repressed the MAO-A activity, but none of the tested urolithins displayed strong inhibitory activity against MAO-B [146]. Similarly, UA inhibited MAO-A and tyrosinase, acted as a free-radical scavenger, and improved the physiological antioxidant defense system in Neuro-2a cells [104].

### 5.3. Other Mechanisms of Action of the Urolithins

Urolithins have been revealed to modulate cell cycle, upregulate tumor suppressor pathways, inhibit proliferation and induce apoptosis in many in vitro experiments on cancer lines including colorectal, liver, pancreas, kidney, and bladder cancer.

In a recent study, UA treatment at concentrations consistent with those found in the intestine triggered autophagy in the human colorectal cancer (CRC) cell line SW620, thereby inhibiting cell survival and metastasis [147]. UA also induced apoptosis in the CRC cell lines HT29, SW480, and SW620 by increasing the expression of proapoptotic proteins p21 and p53 and decreasing the anti-apoptotic protein expression of Bcl-2. Additionally, UA stimulates ROS production and disturbs cellular oxidation status in CRC cells, which can lead to cellular apoptosis and cell death. In contrast, UA treatment did not affect normal human fibroblast cells used as normal control [148]. Previously, UA was shown to upregulate p21 [149] and to inhibit the growth and progression of colon cancer cells and the glycosylation in a p53-dependent manner [150]. Abnormal glycosylation was noticed in major diseases, including cancer; thus, the inhibition of this process could be a potential strategy to prevent tumor cell progression. UD-related glycosylation inhibition in HCT116, SW480, and RKO colon cancer cells resulted in migration and invasion downregulation [151].

UA treatment revealed anti-proliferation in HCT-116 cells through senescence induction associated with the upregulation of p21 and p53 expressions. Moreover, UA and urolithin metabotype B reduced colony formation via the inhibition of cell cycle progression at the G_2_/M phase [152].

The anti-proliferative and anti-invasive effects of UA were also demonstrated in hepatitis B virus (HBV)-positive hepatocellular carcinoma (HCC) [153]. Data showed that UA suppressed the expressions of the proteins Sp-1, widely overexpressed in neoplasms, and Lin28a, the transcriptional target of Sp-1 that could elevate the levels of certain cancer-related miRNAs. Moreover, UA elevated the expression of microRNA let-7a, which functions as a tumor suppressor and is biologically deleted in several cancers, including in HCC patients with HBV infection. UB also exposed the anti-proliferative properties of HCC cells via increasing phosphorylated β-catenin expression and inhibiting Wnt/β-catenin signaling [154].

Another mechanism for UA-induced anti-proliferative and proapoptotic effects was through downregulation of the PI3K/AKT signaling and mTOR pathways. mTOR, a serine/threonine kinase, contributes to cancer cell growth and survival, while PI3K signaling activation is associated with poor prognostic in cancer patients [155]. In pancreatic ductal adenocarcinoma (PDAC) cell lines, UA simultaneously inhibited PI3K/Akt and mTOR activation and mediated the anti-tumor activities, with minimal impact on normal pancreatic epithelial cells [156]. Knowing the extensive crosstalk between PI3K/Akt/mTOR and MAPK pathways, a dual combined reduction of both pathways should be considered for better therapeutic efficacy in human PDAC cell lines [157].

Through downregulation of the PI3K/Akt/mTOR pathway, UA inhibited the proliferation and migration of PDAC cells and increased apoptosis. Even EA, the upstream compound of UA, blocked the cell cycle and reversed epithelial to mesenchymal transition in PDAC by restraining several carcinogenic pathways activated in PDAC, such as NF-κB, COX-2, and Wnt [158].

UA could be a promising therapeutic candidate for cholangiocarcinoma (CCA), the second most common primary hepatic malignancy. In a study on the human intrahepatic CCA cell lines HuCCT-1 and SSP-25, UA treatment-induced G2/M phase cell cycle arrest, thus repressing cell proliferation, and exerted anti-tumor effects by suppressing the Akt/WNK1 signaling pathway and inducing autophagy [159].

The anti-proliferative effect of UA was also demonstrated in the UMUC3 bladder cancer cell line. UA treatment could inactivate the cyclin B1/cdc2 kinase complex and additionally lessened phosphorylation of Akt and ERK, thus downregulating PI3K/Akt signaling pathway [160].

## 6. In Vivo Preclinical and Clinical Evidence for Beneficial Effects of ETs, EA, and Urolithins on Gut–Brain Axis

In order to capture the most relevant results of recent research studies, in this manuscript, we have focused our attention on in vivo original articles and trials published in the last 5 years that revealed the effects of ETs, EA, and urolithins on the gut–brain axis.

### 6.1. In Vivo Studies

#### 6.1.1. In Vivo Studies Performed at the Intestinal Level

Table 2 summarizes the relevant in vivo studies selected for this review highlighting the antioxidant, anti-inflammatory, and anti-tumor effects of ETs, EA, and/or urolithins in the gut.

Xu et al. [161] highlighted the benefits of EA treatment on the intestinal health of C57BL/6J mice. EA stimulated the increase in jejunal villus height (in a dose corresponding to 0.3% from the daily diet, after 21 days of daily treatment), promoted digestion by enhancing the enzymatic activities for the jejunal lactase and sucrase (even at 0.1% EA from daily diet), exercised significant antioxidant effects by stimulating the Nrf2 signaling pathway and positively regulated intestinal microbiota. In an animal model of diquat-induced oxidative stress in C57BL/6J mice, Zhang et al. [171] also found that EA treatment mitigated jejunum oxidative stress via the Nrf2 signaling pathway.

The protective effects of EA also extend to the ileum, as demonstrated by Chen et al. [162]. In an animal model of castor oil-induced diarrhea, EA exercised antioxidant and anti-inflammatory effects and improved mouse immunity by activating the PPAR signaling pathway. The involvement of this signaling pathway in the effects of EA was demonstrated using a PPAR antagonist, GW9662, which inhibited both the antidiarrheic and anti-inflammatory effects of EA. Moreover, RNA sequencing and qRT-PCR revealed that EA treatment increased the expressions of PPAR-gamma and decreased the expressions of inflammatory biomarkers (IL-1β, IL-6, TNF-α, and NF-κB), confirming the outcomes observed in vitro. 

The ability of EA to modulate different signaling pathways was noticed by Lu et al. [163] by transcriptome sequencing in healthy weaned piglets (30 days old). After 40 days of repeated treatment (500 mg/kg), the antidiarrheic and intestinal barrier-stabilizing effects of EA were correlated with the expressing modulation of 401 genes in the jejunal mucosa tissue. Among the downregulated genes (238), genes belonging to seven signaling pathways involved in the immune response were highlighted, underlining the anti-inflammatory potential of EA through multiple biological pathways. In addition, EA promoted the proliferation of IPEC-J2 jejunal enterocytes, increased the expression of Zonula occludens-1 (ZO-1) and Occludin proteins implicated in the tight junction structure of enterocytes and intestinal permeability and promoted a favorable gut-intestinal microbiota in the cecum and rectum. Thus, EA has an important role in protecting the intestinal mucosal barrier function.

Parisio et al. [165] compared the effects of an extract of pomegranate mesocarp (300 mg/kg) with the equivalent amount of the component ellagitannins (45 mg/kg), respectively with an equal amount of lipopolysaccharide components (300 mg/kg), on colitis-induced abdominal pain in rats. All three preparations tested showed antinociceptive effects after 14 days of daily administration, reduced the total amount of mast cells in the colon, decreased the number of degranulated mast cells, and decreased the density of collagen fibers in the colonic mucosal stroma. Through such anti-inflammatory effects, ETs and their metabolites (EA, urolithins) are positively involved in regulating intestinal permeability, controlling the function of the intestinal barrier mucosa, and maintaining tight junctions between epithelial cells. Moreover, through their anti-inflammatory activity, ETs showed significant efficacy in reducing chronic visceral pain related to irritable bowel syndrome or inflammatory bowel diseases.

Fotschki et al. [166] demonstrated that the effects of ETs may be different depending on their type. Thus, monomeric ellagitannins (ME) generate higher levels of bioactive metabolites in the cecum and urine than dimeric ellagitannins (DE). Both ME and DE reduced serum total cholesterol (TC), LDL, and triglycerides (TG) levels and liver oxidized glutathione (GSSG) concentration in rats fed with high-fructose diets and increased the GSH:GSSG ratio, decreased lipid peroxidation in some tissues and revealed blood serum anti-inflammatory activity. These effects were more significant for ME, while DE significantly reduced local microbial activity.

Infections of the digestive tract or pelvic region are usually associated with dysbacteriosis and inflammatory status. Li et al. [167] studied the effects of three polyphenols: EA, gallic acid, and syringin, both individually and in binary associations and then as a tertiary mixture, in a pathogen-induced pelvic inflammatory disease (PID) model in female rats. Acting synergistically, the tertiary mixture exerted the most intense anti-inflammatory effect. The anti-inflammatory action was boosted through controlling apoptosis, activation, and downregulation of the anti-apoptotic BCL-2 and proapoptotic BAX proteins, respectively, and by inhibiting the stimulation of JNK and p38 MAPK, thus regulating the NF-κB signaling pathway. Similarly, UA treatment reduced the abundance of the apoptotic epithelial cells in the colon of mice infected with *Campylobacter jejuni* and attenuated the proinflammatory immune responses in the intestinal tract as well as in the extraintestinal compartments [172]. 

A pomegranate peel extract containing 11% EA-activated caspase-3, an apoptosis executor, in a colonic inflammation model induced by dextran sodium sulfate (DSS) in mice, in a dose corresponding to 26 mg EA/kg bw, which is considered to be the effective anti-inflammatory dose of EA, according to the literature [164]. Nevertheless, EA from pomegranate peel extract was shown to increase the expression of the Bax/Bcl-2 ratio and induce cancer cell apoptosis [173]. Moreover, the pomegranate peel extract could enhance the expression of caspase-9 and induce apoptosis via the mitochondrial pathway [174]. Additionally, UA treatment exerted anti-tumor effects in a rodent model by inhibiting the Akt/WNK1 signaling pathway and inducing autophagy in cholangiocarcinoma cells [159]. These data are particularly important because they make pomegranate a possible and promising candidate in the targeted treatment of cancer. Thus, Lo et al. [170] have already tested the efficiency of a combination of EA and miR-125 nanoparticles to reduce tumor growth through the modulation of the mitochondrial dysfunctions and energetic metabolism of cancer cells.

In a high-fat diet (HFD) model in rats, rich ET chestnut bark extracts normalized intestinal contractility and exerted antioxidative and antiadipose activities suggesting a potential approach to overweight-related diseases [168]. Insulin resistance, one of the consequences of HFD-induced dysbiosis, could be prevented by the synergistic action of EA and certain probiotics such as *Weizmannia coagulans*. In addition, the combination of EA and *W. coagulans* BC2000 inhibited HFD-induced endotoxemia and activated the hepatic autophagy pathway in a manner similar to that of urolithin [169].

#### 6.1.2. In Vivo Studies Performed at the Cerebral Level

Several recent studies have shown a strong correlation between gut microbiota dysbiosis and inflammatory processes both in the gastrointestinal tract and extraintestinal compartments [163,172]. The prebiotic role of polyphenols, which have the ability to modulate gut microbiota, is well known. As already stated, their metabolites formed in the gut under the action of the microbiota, considered as metabolites of the microbiota, are bioactive compounds with important biological actions for the health of the host organism and the maintenance of its homeostasis [71]. ETs and EA, as well as urolithins, are the focus of many researchers due to their antioxidant, anti-inflammatory, and anti-cancer effects, as well as the anti-atherogenic, neuroprotective, pro-mitophagy, etc., effects, and they are currently the molecules of pharmaceutical interest.

Table 3 summarizes recent studies selected from the literature highlighting the impact of different ETs, EA, urolithins, or natural sources (extracts or juices) rich in ETs, on the gut–brain axis, with important benefits for brain health and neuronal processes.

Aging is a complex process involving multiple pathophysiological events, such as oxidative stress, chronic low-intensity inflammation called “inflamm-aging”, mitochondrial dysfunctions, impaired protein homeostasis, and epigenetic mechanisms [88]. D-galactose (D-gal) is used experimentally in murine models of aging, because in high doses it induces free radicals and ROS, and oxidative stress, which accelerates senescence, including the brain [195]. Thus, Chen et al. tested the anti-aging potential of EA [175], UA [176], and UB [145] in murine models of aging obtained by i.p. injection of D-gal (100–150 mg/kg/day) for 8 weeks. EA (150 mg/kg/day, per os (p.o.)) clearly showed anti-aging potential, antioxidant and anti-inflammatory effects as well as hepatoprotective and neuroprotective properties against the toxicity of chronic exposure to high doses of D-gal. EA could prevent or attenuate the progression of aging-induced structural changes in the brain and liver by modulating the expression of some aging-related proteins [175]. UA (150 mg/kg/day, p.o.) showed evident neuroprotection, kept the morphological structure of neurons in the CA3 region of the hippocampal tissue, improved the cognitive functions impaired by D-gal, exercised antioxidant effects in the brain, and modulated the neuromediator levels by decreasing acetylcholinesterase (AChE) and MAO levels. The neuroprotective and anti-aging effects of UA are based on the regulation of autophagy through activation of the miR-34a-mediated SIRT1/mTOR signaling pathway [176]. UB (150 mg/kg/day, p.o.) also promoted neuronal survival, ameliorated neurological deficits and cognitive functions in D-gal-induced aging mice, and protected brain against oxidative stress. UB can be considered, together with UA, as brain health care products for the prevention of age-related neurodegenerative diseases such as AD or PD [145].

The development of AD is related to genetic factors but also depends on oxidative stress conditions and neuroinflammation [178]. EA has been tested in various animal models of AD or cognitive impairment at doses of 50–100 mg/kg body weight (bw)/day administered orally or parenterally. Thus, Zhong et al. [177] investigated the neuroprotective potential of EA (50 mg/kg/day) in APP/PS1 double-transgenic mice vs. wild-type (WT) C57BL/6 mice after repeated oral administration for 60 days. EA suppressed Aβ production and reduced Aβ deposition in the brain of APP/PS1 mice, and ameliorated the cognitive deficits of these animals in Morris water maze (MWM) test. In the same time, EA treatment activated threonine-protein kinase (Akt) and downregulated the activity of glycogen synthase kinase (GSK)3β, thereby reducing the tau protein hyperphosphorylation in the hippocampus by the RAC-α serine/Akt/GSK3β signaling pathway. UA (300 mg, p.o., 14 days) also decreased the Aβ plaque accumulation in the cortex and hippocampus of APP/PS1 mice, mitigated the neuroinflammation via reducing reactive gliosis and downregulating the AMPK/NFκB and AMPK/p38MAPK inflammatory signaling pathways, activated the hippocampal neurogenesis and reduced the neuronal apoptosis [181]. In a streptozotocin (STZ)-induced diabetic mouse model, parenteral UA treatment (UA—2.5 mg/kg/day, i.p., 8 weeks) was also efficient to suppress high glycemia-induced Aβ neuronal amyloidogenesis and tau hyperphosphorylation. The molecular mechanisms involved were the decreasing of mitochondrial calcium influx and mitochondrial ROS accumulation, and downregulation of amyloid precursor protein (APP) and β-secretase-1 (BACE1) expressions. UA also reversed the high glucose-activated AhR signaling and suppressed the transglutaminase type 2 (TGM2) expression [143]. Moreover, UA (2.5 mg/kg/day, i.p., 3×/week, 4 months), showed protective effects against human Aβ peptide-induced toxicities in an animal model of late-onset AD using humanized homozygous amyloid beta knockin (hAbKI) mice, both alone and in combination with EGCG (25 mg/kg/day). The phenotypic behavior (including motor coordination, locomotor abilities, working memory, and spatial learning and memory) was significantly enhanced, mitochondrial biogenesis improved, synaptic, mitophagy, and autophagy genes upregulated, and mitochondrial dysfunctions significantly reduced. The combined therapy UA + EGCG was more effective and stronger than UA alone [182].

Ramadan et al. [178] tested EA (50 mg/kg) to assess its effects on the episodic memory (by the novel object recognition test (NORT)) and the changes in the entorhinal cortex (ERC) in an AD Wistar rat model induced with AlCl_3_. The ERC is the most affected cortical area in AD patients and its progressive deterioration is involved in early memory loss. By the antioxidant activity, EA treatment restored the discrimination index in NORT test and reduced the structural changes in the ERC sections in AD rats, thus restoring episodic memory.

The sleep disorder (SD) could affect neurobehaviors, including cognition and memory. EA (50 mg/kg and 100 mg/kg) mitigated the neurobehavioral abnormalities associated with memory impairment and anxiety (in the MWM, NORT, object location, open field (OF), and elevated plus maze (EPM) tests) in SD mice after i.p. administration for 21 days. The EA treatment ensured neuron survival and the high dendritic spine density in the hippocampus of SD mice by reducing oxidative stress via the Nrf2/ARE pathway activation and by inhibiting the neuroinflammation via Toll-like receptor 4 (TLR4) downregulation [179].

In an animal model of brain inflammation induced by cerebral ischemia/reperfusion (I/R) performed in Wistar rats, Falahieh et al. [180] found that EA (100 mg/kg/day, p.o.) restored the blood-brain barrier (BBB) permeability, significantly reduced the neuroinflammation and brain edema, and improved all neurological signs scores and neurobehavioral abnormalities (in EPM, OF, and the forced swimming tests). In a mouse model of traumatic brain injury, UA (2.5 mg/kg, i.p.) treatment significantly reduced brain edema and protected the tight junction proteins and the BBB function in the injured cortex, thus improving the mouse neurological function. The neuroprotective effect of UA is mediated by its anti-inflammatory activity and autophagy enhancement via the inhibition of the PI3K/Akt/mTOR and Akt/inhibitor of NFκB (IκB) kinase (IKK)/NFκB signaling pathways [192].

These outcomes are very important because they demonstrate the ability of EA or its microbial metabolites, the urolithins, to improve/restore not only the permeability of the intestinal barrier but also the permeability of the BBB affected by pathophysiological processes as cerebral ischemia/reperfusion or traumatic brain injury, and encourage further research to explain the mechanisms of action on the gut–brain axis in more detail.

Moon et al. [183] prepared ethanolic extracts of *Juglans regia* (Gimcheon 1ho cultivar, GC) rich in EA and ETs and tested them in a diabetic mouse model with cognitive dysfunction. GC extracts had the ability to restore the neurobehavioral and memory dysfunction induced by high glycemia (in Y-maze, passive avoidance, and MWM tests). Moreover, GC extracts showed antioxidant effects, anti-inflammatory activity via the JNK/NFκB signaling pathway, suppressed synaptic disorders by regulating the cerebral cholinergic system, regulated mitochondrial activity and neuronal apoptosis pathway.

PD is another neurodegenerative disease that develops progressively under conditions of oxidative stress and neuroinflammation. It is characterized by loss of dopaminergic neurons and dopamine depletion in the midbrain substantia nigra pars compacta (SNpc), motor and olfactory dysfunctions, and the accumulation of the misfolded α-synuclein that forms Lewy bodies [184]. The beneficial effects of EA and ETs in PD have been shown in preclinical studies in various animal models. Thus, Kujawska et al. [184,185] tested pomegranate juice (PJ) on rats with rotenone-induced PD and found that the pretreatment with PJ followed by simultaneous exposure to rotenone and PJ provides neuronal protection from oxidative stress, enhanced the activity of mitochondrial aldehyde dehydrogenase, normalized the expression of anti-apoptotic Bcl-xL protein, prevents accumulation of α-synuclein and dopamine (DA) depletion in the midbrain, and improved olfactory function. They also highlighted the presence of UA in the brain and its possible involvement in the neuroprotective action of PJ.

Tancheva et al. [186] tested three antioxidant compounds: EA (50 mg/kg), α-lipoic acid (LA), and myrtenal (Myrt), on rats with PD induced with 6-hydroxydopamine (6-OHDA) intrastriatal injected. All compounds showed a significant antiparkinsonian effect, improved neuromuscular coordination, learning, and memory performance (in apomorphine-induced rotation, rotarod, and passive avoidance tests), and restored dopamine levels impaired by 6-OHDA injection. DA neuronal loss experimentally induced with LPS in rats was significantly mitigated by EA (50 mg/kg). The mechanism involved was a significant reduction of neuroinflammation via the suppression of microglial NLRP3 inflammasome signaling activation. The neuroprotective properties of EA were also reflected by the reduction of tyrosine hydroxylase levels in substantia nigra neurons [187].

MS is characterized by inflammatory cell infiltration and demyelination in the CNS [190]. Two studies performed on EAE in mice immunized with MOG_35–55_ showed the ability of EA (50 mg/kg/day), a pomegranate peel extract (PEm) rich in EA and punicalagin [191], and of UA (25 mg/kg/day) [190], respectively, to suppress the progress of EAE and to ameliorate the clinical symptoms. A significant reduction of microglia activation and astrocytosis, particularly in the gray matter of the spinal cord, as well as a reduction of the CD45 staining at this level, suggest the potential therapeutic benefit of EA or PEm treatment in MS [191]. UA significantly reduced infiltrating mononuclear cells in CNS, but its effect on the immune response was of mild intensity at the periphery [190]. These studies highlighted the real potential of ETs, EA, and/or UA in the treatment of autoimmune diseases such as MS.

EA (100 mg/kg) [188] and corilagin (10 mg/kg and 20 mg/kg), an ET found in pomegranate leaves, but also in other medicinal plants [189], modulated seizure susceptibility in pentylenetetrazole (PTZ)-induced animal models of seizures.

Oenothein B, a dimeric ET widely distributed in various medicinal plants, has a hydrophilic structure and may not have been able to pass through the BBB. Okuyama et al. [193] found that oenothein B activated neurotrophic factors in the hippocampal region of healthy mice: ERK2 and cAMP response element-binding protein (CREB), known as an important regulator of the expression of brain-derived neurotrophic factor (BDNF). Thus, its neuroprotective effects are mediated through not only its anti-inflammatory activity but also by the activation of some neurotrophic factors in the brain.

Neurotoxoplasmosis, a chronic infection with *Toxoplasma gondii* (*T. gondii*) characterized by the persistence of parasite cysts in the brain, is associated with high mortality in immunocompromised patients and with poor tolerability of the current therapy, which is not effective against parasite cysts. UA was tested and has been shown to be effective in the treatment of mice chronically infected with *T. gondii*, reducing the parasite cyst formation and significantly prolonging survival. Thus, UA is a promising natural bioactive compound that can be used in the treatment of neurotoxoplasmosis [194].

### 6.2. Clinical Studies

Oxidative stress and inflammatory conditions play a major role in the pathogenesis of irritable bowel syndrome (IBS). In a double-blind, placebo, parallel randomized clinical trial (RCT) in patients with IBS (22 EA vs. 22 placebo), the intake of 180 mg of EA per day for 8 weeks reduced abdominal pain and distention, flatulence, and rumbling [196]. Moreover, EA consumption significantly ameliorated the quality of life (QOL), increased total antioxidant capacity (TAC), and lowered MDA, C-reactive protein (CRP), and IL-6 levels, corresponding to an attenuated overall score of IBS-QOL [197]. These changes were not detected in the placebo group.

In a double-blind, randomized, placebo-controlled trial conducted in middle-aged and older adults (98 PJ vs. 102 placebo), daily consumption of PJ (236.5 mL) for 1 year kept stable the ability to learn visual information vs. the significant decline observed in the placebo group [198].

In another double-blind, parallel, placebo-controlled trial (11 PJ vs. 9 placebo), stool and plasma were collected at baseline and after 1 year of PJ consumption and analyzed to investigate the tryptophan (Trp) metabolism. Plasma level of indole propionate (IPA), a microbial metabolite of Trp, was significantly decreased in the placebo group and it was kept stable in the PJ-treated group at the end of the study vs. baseline. Other major metabolites of Trp (serotonin, kynurenine, and indole acetate), as well as the Trp levels, did not change significantly in either group. PJ consumption was associated with a reduction of the abundance of two genera, *Shigella* and *Catenibacterium*, negatively correlated with plasma IPA levels. These outcomes were also confirmed in vivo. Thus, the microbial Trp metabolism could contribute to the health benefits of ETs [199]. 

Aging is associated with a decline in mitochondrial function. Several recent clinical trials have looked at the effects of long-term high-dose UA supplementation on mitochondrial and cellular health in middle-aged and older adults [200,201,202]. Healthy mitochondria play a critical role in cell function and homeostasis, and impaired mitochondrial function is implicated in the onset and development of several neurodegenerative diseases [202].

Andreux et al. [202] conducted the first placebo-controlled, double-blind RCT with different doses of UA (250, 500, 1000, or 2000 mg) administered for 28 days in healthy, sedentary elderly. The results demonstrated that UA was an activator of mitophagy and induced mitochondrial gene expression in skeletal muscle.

In an RCT by Liu et al. [201], 66 adults aged 65–90 years received 1000 mg UA/day. After 4 months, plasma levels of some biomarkers of mitochondrial health, such as several acylcarnitines, ceramides, as well as CRP levels, were decreased compared to placebo. These effects were clinically correlated with a significant improvement in muscle endurance vs. placebo. Similar results were obtained by Singh et al. [200] in a placebo-controlled, double-blind RCT with 88 middle-aged adults.

Therefore, these studies certify that UA supplementation is safe and it generates increased circulating levels of UA. UA is a promising therapeutic agent with real anti-aging potential and could be used in the prevention of age-related muscle decline. Furthermore, oral administration of chemically synthesized UA is safe in humans, and 1000 mg/serving could be used as a functional food ingredient [202]. Moreover, a recent in silico analysis estimated that EA was not mutagenic or carcinogenic, had low toxicity, and showed anti-inflammatory and antimicrobial activities [203].

## 7. Conclusions

ETs and EA are bioactive polyphenols that are found in several plant-source foods, including walnuts, berries, and pomegranates. Urolithins, their gut microbiota-derived metabolites, are better absorbed and might be responsible for the beneficial effects ascribed to ETs and EA.

In this review, we analyzed the last recent-published findings on ETs, EA, urolithins and the intestinal and brain effects, the potential mechanisms of action and the connection between the ET microbiota metabolism and the consequences detected on the gut–brain axis.

Our review highlights much preclinical and clinical evidence indicate that attests that ET-rich foods associated with individual gut microbiome or certain urolithins, especially UA and UB, could promote beneficial health impact. Their biological actions are complex, including the modulation of many important signaling pathways involved, particularly in inflammation and aging, as well as the function stabilization of the intestinal barrier and BBB. A better understanding of the role of these metabolites in the disease pathogenesis may assist in the prevention or treatment of pathologies targeting the gut–brain axis. Based on our knowledge, this review is the first one to focus on the impact ETs, EA, and urolithins could have on the gut–brain axis.

However, future preclinical detailed toxicological evaluations and clinical investigations of individual gut microbiota composition and health status in different age groups should be conducted before the therapeutic application of EA-enriched foods in human population.

## Figures and Tables

**Table 1:** Contents of ellagitannins and ellagic acid in fruits, nuts, and beverages.

Food Product	ETs/EA	Content	Reference
Fruits			
Raspberry fruit (*Rubus idaeus* L.)	Sanguiin H-10	0.62 mg/g dw *	[26]
	Sanguiin H-6	9.56 mg/g dw	[26]
	Lambertianin C	9.79 mg/g dw	[26]
	Ellagic acid	0.31 mg/g dw	[26]
Blackberry fruit (*Rubus fruticosus* L.)	Lambertianin C	11.0 mg/100 g rw **	[30]
	Lambertianin A	39.8 mg/100 g rw	[30]
	Ellagic acid	11.8 mg/100 g rw	[30]
Strawberry (*Fragaria* × *ananassa* Duch.)—fruits of six cultivars	Pedunculagin	0.24–1.38 mg/g dw	[29]
	Potentillin	0–1.69 mg/g dw	[29]
	Casuarictin	0.12–1.30 mg/g dw	[29]
	Sanguiin H-6	0.12–1.55 mg/g dw	[29]
	Agrimoniin	0.89–13.11 mg/g dw	[29]
	Fragariin A	0.34–1.47 mg/g dw	[29]
Pomegranate (*Punica granatum* L.)—peels of seven cultivars	Pedunculagin	8.2–11.8 mg/g dw	[27]
	α-punicalagin	17.4–24.7 mg/g dw	[27]
	β-punicalagin	25.3–32.9 mg/g dw	[27]
	β-punicalin	3.1–7.1 mg/g dw	[27]
	Ellagic acid hexoside	2.9–6.3 mg/g dw	[27]
	Galloyl-HHDP-gluconic acid	1.1–1.7 mg/g dw	[27]
	Galloyl-HHDP-hexoside	1.0–2.8 mg/g dw	[27]
	HHDP-hexoside	1.7–3.2 mg/g dw	[27]
	Ellagic acid	2.1–5.3 mg/g dw	[27]
Mango peel and seed (*Mangifera indica* L.)	Valoneic acid dilactone	Not mentioned	[59]
	Ellagic acid	1.13–13.67 mg/100 g dw	[60]
Costa Rican guava peel and flesh (*Psidium* *friedrichsthalianum* Nied.)	Geraniin isomer 1	120.6 mg/100 g dw (peel, flesh)	[28]
	Geraniin isomer 2	9.3 mg/100 g dw (peel); 103.9 mg/100 g dw (flesh)	[28]
	Castalagin isomer 1	28.3 mg/100 g dw (peel); 39.6 mg/100 g dw (flesh)	[28]
	Castalagin isomer 2	84.6 mg/100 g dw (peel); 41.7 mg/100 g dw (flesh)	[28]
Muscadine grapes (*Vitis rotundifolia* Michx.)	Sanguiin H-5	0.03–0.91 mg/g	[48]
	Ellagic acid	32.1 mg/100 g fw ^#^ (seed); 59.1–61.5 mg/100 g fw (skin); 14.63–17.70 mg/100 g fw (fruit)	[61]
Jabuticaba (*Myrciaria cauliflora* Mart.)	Castalagin	78.4 mg/100 g fruit fw	[62]
	Vescalagin	28.7 mg/100 g fruit fw	[62]
	Pedunculagin	9.8 mg/100 g fruit fw	[62]
Yellow grumixama fruit (*Eugenia brasilienses* Lam.—γ variety)	Castalagin/vescalagin, pedunculagin, strictinin, potentillin/casuarictin	92 mg EAE ^##^/100 g fw	[63]
Purple grumixama fruit (*Eugenia brasilienses* Lam.—α variety)	Pedunculagin, strictinin, potentillin/casuarictin, tellimagrandin I	82−243 mg EAE/100 g fw	[63]
Camu-camu (*Myrciaria dubia* (Kunth) McVaugh)	Alnusiin	Not mentioned	[53]
Java plum/black plum fruit (*Syzygium cumini* (L.) Skeels)	Vescalagin	0–1.0 mg/g dw	[64]
	Galloyl dihexahydroxydiphenoyl glucose	1.0–7.5 mg/g dw	[64]
	Digalloyl dihexahydroxydiphenoyl glucose	0.97–5.6 mg/g dw	[64]
	Ellagic acid	1.1–11.9 mg/g dw	[64]
Nuts			
Walnuts (*Juglans regia* L.)—pellicle of six cultivars	Pedunculagin/casuariin (2 isomers)	3.1–13.3 mg/g fw	[31]
	Strictinin/isostrictinin (3 isomers)	1.9–9.6 mg/g fw	[31]
	Trigalloyl-HHDP-glucose (4 isomers)	2.9–27.5 mg/g fw	[31]
	Tellimagrandin 1 (3 isomers)	3.1–27.9 mg/g fw	[31]
	Pterocarinin A	0.5–2.8 mg/g fw	[31]
	Castalagin/vescalagin	9.5–35.9 mg/g fw	[31]
	*bis*-HHDP-glucose derivative	20.2–26.1 mg/g fw	[31]
	Casuarin/casuarictin	8.6–39.8 mg/g fw	[31]
	Ellagic acid	17.5–23.3 mg/g fw	[31]
Pecans (*Carya illinoinensis* (Wangenh.) K.Koch)	Valoneic acid dilactone	9.45 μg/g acetonic crude extract	[65]
	Ellagic acid	132.0 μg/g acetonic crude extract	[65]
Chestnut (*Castanea sativa* Mill.)—peels of cultivar Longal	Vescalagin	67.5–109.4 μg/g dw	[32]
	Castalagin	49.6–100.4 μg/g dw	[32]
	Ellagic acid	47.6–3542.6 μg/g dw	[32]
Beverages			
Red oak-aged wines	Castalagin	0.61–11.8 mg/L	[57]
		0.07–11.43 mg/L	[17]
	Vescalagin	0.194–6.4 mg/L	[57]
		0.06–1.32 mg/L	[17]
	Grandinin	0.36–3.4 mg/L	[57]
		0.02–0.63 mg/L	[17]
	Roburin A	0.01–0.24 mg/L	[17]
	Roburins B + C	0.07–1.56 mg/L	[17]
	Roburin D	0.04–0.91 mg/L	[17]
	Roburin E	0.115–2.08 mg/L	[57]
		0.02–0.79 mg/L	[17]
	Ellagic acid	7.88–11.61 mg/L	[66]
White oak-aged wines	Castalagin	0.328 mg/L	[57]
	Vescalagin	0.14 mg/L	[57]
	Grandinin	0.061 mg/L	[57]
Rosé oak-aged wines	Castalagin	4.69 mg/L	[57]
	Vescalagin	2.3 mg/L	[57]
	Grandinin	3.5 mg/L	[57]
	Roburin E	1.5 mg/L	[57]
Cognac eaux-de-vie	β-1-*O*-ethylvescalagin	1.1 mg/L	[18]
	β-1-*O*-ethylvescalin	0.73–4.66 mg/L	[18]
	Brandy tannin A	0.77–10.28 mg/L	[18]
		0.4–4.2 mg/L	[67]
	Brandy tannin B	0.5–5.50 mg/L	[18]
	Castalagin	1.34–4.90 mg/L	[18]
	Vescalagin	0.06–0.64 mg/L	[18]
	Grandinin	0.11–1.50 mg/L	[18]
	Roburin A	0.01–0.07 mg/L	[18]
	Roburin B	0.01–0.17 mg/L	[18]
	Roburin C	0.03–0.61 mg/L	[18]
	Roburin D	0.26–1.48 mg/L	[18]
	Roburin E	0.03–0.61 mg/L	[18]
Commercial cognac	β-1-*O*-ethylvescalin	0.87–1.58 mg/L	[18]
	Brandy tannin A	1.7–3.38 mg/L	[18]
		0.03–7.7 mg/L	[67]
	Brandy tannin B	0.37–1.21 mg/L	[18]

* dw—dry weight; ** rw—raw material; ^#^ fw—fresh weight; ^##^ EAE—ellagic acid equivalents (total ellagitannin content expressed as total ellagic acid); HHDP—hexahydroxydiphenic acid.

**Table 2:** Preclinical studies attesting the antioxidant, anti-inflammatory, and anti-tumor effects of ellagitannins, ellagic acid, or their metabolites at the intestinal level.

Substances Tested (ST)—Doses	Preclinical Model	Main Results	Reference
EA	C57BL/6J mice were divided into 3 groups: control, standard pellet diet supplemented with 0.1% EA, and with 0.3% EA, respectively, for 21 consecutive days.	EA treatment increased jejunal villus height (0.3% EA) and enhanced the enzymatic activities for the jejunal lactase and sucrase (both 0.1% and 0.3% EA), and the alkaline phosphatase (0.3% EA). EA (0.3%) showed significant antioxidant effects by increasing the mRNA expression of Nrf2 and HO-1, the enzymatic activities of the superoxide dismutase and catalase, and reducing the malondialdehyde level in the jejunum. EA (0.3%) proved a suitable ability to regulate intestinal microbiota: increased the count of *Lactobacillus* species and decreased the count of *Escherichia coli*.	[161]
EA—10 mg/mL	Castor oil-induced diarrhea in mice. BALB/c mice were divided into 4 groups: (1) control; (2) castor oil (orally, 0.2 mL); (3) castor oil (orally, 0.2 mL) + EA (orally, 0.3 mL) after 30 min; (4) 0.1 mL GW9662 (i.p., 1 mg/mL) and castor oil (orally, 0.2 mL) + EA (orally, 0.3 mL) after 30 min.	Transcriptome, histological assay, and qRT-PCR were performed on ileum tissues. EA protected the ileum of mice against castor oil-induced diarrhea, reducing inflammation. The pretreatment with GW9662, a PPAR-specific antagonist, inhibited the anti-inflammatory effect of EA.	[162]
EA-supplemented feed (500 mg/kg)	Healthy weaned piglets (30 days old) received feed supplemented with EA twice daily for 40 days vs. a control group fed with the same feed without EA.	EA reduced the diarrhea rate, significantly increased weight gain, and diminished the serum diamine oxidase (DAO) levels of weaned piglets. Transcriptome sequencing revealed the ability of EA to down-regulate the expression of some genes involved in seven pathways related to immune response. EA modulated the microbiota composition in the cecum and rectum.	[163]
Pomegranate peel extract (PPE) containing 11% EA	A colonic inflammation model induced by dextran sodium sulfate (DSS) in mice (7-day cycles with 2% DSS in drinking water and 7 days of drinking water without DSS, for 42 days). Swiss Webster mice receiving 2% DSS were divided into 5 groups: control, PPE-dose 1 (240 mg/kg/day), PPE-dose 2 (480 mg/kg/day), ASP (43 mg/kg/day), and ASP (43 mg/kg/day) + EA (26 mg/kg/day).	PPE (dose 2) significantly increased the caspase-3 expression in mice colon tissues collected three days after the last treatment of DSS.	[164]
Pomegranate mesocarp decoction (PMD)—300 mg/kg; Polysaccharide components of PMD (PCs)—300 mg/kg; Ellagitannin components of PMD (ECs)—45 mg/kg	Colitis-induced abdominal pain in male Sprague–Dawley rats by 2,4-dinitrobenzenesulfonic acid (DNBS). ST was orally administered for 14 days.	ECs have been more effective in reducing visceral pain than the equivalent dose of PMD, both at 7 and 14 days of repeated administration. All three treatments significantly diminished the inflammation degree of the colonic mucosa and the fibrosis state. However, ECs are the responsible bioactive compounds of PMD, and PCs support and enhance their effects.	[165]
Two dietary strawberry extracts rich in monomeric ellagitannins (ME) and dimeric ellagitannins (DE), respectively	Male Wistar rats fed with high-fructose diets for 6 weeks: 3 groups received a diet based on corn starch (C): C, C + ME, C + DE, in parallel with other 3 groups fed with a diet containing fructose (F): F, F + ME, F + DE.	ME was more effective in reducing oxidative stress (lipid peroxidation in some tissues) and serum inflammatory biomarkers (TNF-α, IL-6), as well as the serum and liver triglyceride levels, than DE. Higher levels of ET metabolites were determined in the cecum and urine of rats receiving ME than of those fed with DE. Both ME and DE regulated the biochemical disturbances induced by a high-fructose diet. The efficacity of the ME extract was associated with systemic parameters, while that of the DE extract was associated with local microbial activity.	[166]
Feiyangchangweiyan capsule (FYC) and its main components: EA, gallic acid (GA), and syringin (SY)	A pathogen-induced pelvic inflammatory disease (PID) model in female SD rats (E. coli and S. aureus induced infection in the upper genital tract) with 11 groups: control, PID, FYC (1.2 g/kg), GA (210 mg/kg), EA (30 mg/kg), SY (35 mg/kg), GA (105 mg/kg) + EA (15 mg/kg), GA (105 mg/kg) + SY (18 mg/kg), EA (15 mg/kg) + SY (18 mg/kg), GA (70 mg/kg) + EA (10 mg/kg) + SY (12 mg/kg), and Fuke Qianjin capsule (FKC) (2.4 g/kg) as a positive control.	Histological analysis, ELISA assays, and Western blot analysis were applied to detect the expression of NF-κB, BAX, BCL-2, and JNK. The expressions of IL-1β, TNF-α, MPC-1, and BAX induced by infection were significantly reduced, while the IL-10 level and the expression of Bcl-2 were increased by FYC, but also by its main components. The anti-inflammatory effects in the rats of the GA + EA + SY group were more intense than those observed in the rats treated with dimers or monomers. PID was associated with an elevated expression of BAX and a dramatic suppression of BCL-2 expression. FYC, and especially EA and SY, have shown an increased efficacy in reversing these effects.	[167]
Extract of Chestnut bark (ENC^®^) (20 mg/kg/day) rich in ellagitannins (administered by gavage)	A high-fat diet (HFD) model in rats Male Sprague–Dawley rats were divided into 4 groups: control (regular diet, RD), HFD, RD + ENC^®^, and HFD + ENC^®^, for 21 days.	In HFD-fed rats, ENC^®^ improved lipidic profile (significantly reduced TC, LDL-C, TG, and increased HDL-C), exerted antioxidative and anti-inflammatory activities, and normalized intestinal contractility in ileal and colonic tissues.	[168]
EA—0.3 g/kg of HFD; *Weizmannia coagulans* BC2000 and BC77—0.1 g of lyophilized powder (containing 4 × 10^11^ CFU/g)/kg feed	Animal model of HFD-induced insulin resistance: C57BL/6J male mice were divided into 5 groups: (1) Low-fat diet (LFD); (2) HFD (providing 60% of fat energy); (3) HFD + EA; (4) HFD + EA + BC77; (5) HFD + EA + BC2000.	EA and *W. coagulans* BC2000 had a synergistic effect in reducing the insulin resistance index and HFD-induced endotoxemia. EA co-administered with BC200 activated the autophagy pathway in the mouse liver, a urolithin-like effect, but not with BC77. *W. coagulans* BC2000 promoted a favorable intestinal environment for the proliferation of EA-transformable bacteria.	[169]
EA (80 mg/kg) and miR-125 (1.25 mg/kg) nanoparticles carrying a mitochondrion-directed peptide (K) and a tumor-targeted ligand (L)	A SAS-tumor bearing mouse model. BALB/c nude mice were divided into 5 groups: saline (control), EA, EA/LPN (lipid-polymer nanoparticles), EA/LPN-KL, and EA/LPN-KL + miR-125/SLN (solid lipid nanoparticles)-KL. The formulations were administered every 2 days for 20 days.	All EA formulations have shown hypoglycemic and hypolipidemic effects, but the combined formula, EA/LPN-KL + miR-125/SLN-KL, was the most effective. This formula also showed great tumor-suppression ability in SAS-tumor-bearing BALB/c mice.	[170]
Urolithin A (UA)—20 mg/kg	Animal model of cholangiocarcinoma (CCA) in xenograft female nude mice (BALB/c Slc-nu/nu) injected with HuCCT-1 cells (5 × 10^6^ cells/100 mL of media). UA treatment was initiated 2 weeks after tumor transplantation for a period of 35 days (orally, 3×/week).	The tumor volume was calculated twice a week, and immunohistochemical analysis was performed at the end of the experiment. UA inhibited tumor growth and increased LC3-II levels. On the other hand, the phospho-kinase array demonstrated the downregulation of the Akt/WNK1 pathway. LC3-II expression was elevated in WNK1 knocked-down cells, indicating that WNK1 is the key signal for regulating autophagy. Thus, UA exerted anti-tumor effects by suppressing the Akt/WNK1 signaling pathway and inducing autophagy.	[159]
EA—100 mg/kg bw	Animal model of oxidative stress induced in C57BL/6J mice by diquat (25 mg/kg bw, single dose). EA was administered orally for 5 days.	EA treatment significantly reduced diquat-induced weight loss and mitigated oxidative stress in jejunum: reduced ROS production, up- regulated the mRNA expression of Nrf2 and the antioxidant enzymes (GPx1 and HO-1). ML385, a specific Nrf2 inhibitor, counteracted the EA effects on jejunum oxidative stress.	[171]
UA (synthetic)—daily dose of 0.114 mg/kg bw in drinking water	Animal inflammation model: microbiota-depleted IL-10−/− mice perorally infected with *C. jejuni* (on days 0 and 1). UA treatment was initiated on day 2 post-infection (p.i.) for 5 consecutively days vs. a placebo group (water).	Versus the placebo group, UA improved the early (<24 h) clinical status of infected mice, reduced pathogen loads in the ileum, immune cell and apoptotic epithelial cell abundance in the colon (by histopathology and immunohistochemistry), and the secretion of proinflammatory biomarkers (IFN-γ, TNF-α, MCP-1, and IL-6) both in the intestinal tract and in the extraintestinal compartments (lung, liver, kidney, serum).	[172]

Akt—protein kinase B; bw—body weight; EA—ellagic acid; GPx1—glutathione peroxidase 1; HDL-C—high-density lipoprotein-cholesterol; HFD—high-fat diet; HO-1—heme oxygenase-1; IFN-γ—interferon γ; IL—interleukin; LDL-C—low-density lipoprotein-cholesterol; MCP-1—monocyte chemotactic protein-1; Nrf2—nuclear factor erythroid 2-related factor 2; ROS—reactive oxygen species; TC—total cholesterol; TG—triglycerides; TNF-α—tumor necrosis factor α; UA—urolithin A.

**Table 3:** Preclinical studies attesting the antioxidant, anti-inflammatory, and neuroprotective effects of ellagitannins, ellagic acid, or their metabolites in the brain.

Substances Tested (ST)—Doses	Preclinical Model	Main Results	Reference
EA—p.o. administered in 3 doses: high (H), medium (M), and low (L) doses	Aging model obtained with D-gal (100 mg/kg/day, s.c., 8 weeks) in male Sprague–Dawley (SD) rats divided into 6 groups: (1) control, (2) D-gal, (3) positive control (vitamin E, 150 mg/kg, by gavage), (4) H-EA (D-gal + 150 mg EA/kg/day), (5) M-EA (D-gal + 100 mg EA/kg/day), (6) L-EA (D-gal + 50 mg EA/kg/day).	EA restored the antioxidant defense system (evaluated by SOD, CAT, GSH-Px, and T-AOC activities, and MDA levels, respectively) in the liver and brain of D-gal-induced aging rats, especially in H-dose. The treatment with M and H doses of EA for 8 weeks has significantly mitigated the D-gal-induced inflammation (TNF-α, IL-6, and IL-1β levels in serum) and the liver function decline (ALT and AST levels). Histopathological analysis showed that the H-dose of EA was more protective and kept the morphological structure in both the liver and brain. EA treatment significantly downregulated the expression of Bcl-2 and Bax proteins and showed anti-apoptotic effects in a concentration-dependent manner.	[175]
UA—p.o. administered in 3 doses: high (H), medium (M), and low (L) doses	I. Aging model obtained with D-gal (150 mg/kg/day, s.c., 8 weeks) in male Institute of Cancer Research (ICR) mice were divided into 5 groups: (1) control (Ctrl), (2) D-gal, (3) H-UA (D-gal + 150 mg UA/kg/day), (4) M-UA (D-gal + 100 mg UA/kg/day), (5) L-UA (D-gal + 50 mg UA/kg/day). II. Additional experiment with 4 groups: - 2 groups of 2-month-old mice: control and UA (150 mg UA/kg/day for 2 months); - 2 groups of 12-month-old mice: control and UA (150 mg UA/kg/day for 2 months).	UA treatment significantly ameliorated D-gal-induced behavioral impairments (in Open field, Morris Water Maze, and Object–Place Recognition tests). UA significantly lowered the AChE and MAO levels and the oxidative stress (the activities of SOD, CAT, GSH-Px, T-AOC, and MDA levels, respectively) in the brain of D-gal-induced aging mice. UA showed neuroprotection against D-gal-induced aging downregulating miR-34a in the hippocampal tissue and activated autophagy by upregulating SIRT1 and downregulating the protein expression of p53/p21 and the mTOR signaling pathway.	[176]
Urolithin B (UB)—i.g. administered in 3 doses: high (H), medium (M), and low (L) doses	I. Aging model obtained with D-gal (150 mg/kg/day, s.c., 8 weeks) in male C57BL/6 mice were divided into 5 groups: (1) control, (2) D-gal, (3) H-UB (D-gal + 150 mg UB/kg/day), (4) M-UB (D-gal + 100 mg UB/kg/day), (5) L-UB (D-gal + 50 mg UB/kg/day). II. Additional experiment with 4 groups: - 2 groups of 2-month-old mice: control and UB (150 mg UB/kg/day for 2 months); - 2 groups of 12-month-old mice: control and UB (150 mg UB/kg/day for 2 months).	Long-term UB treatment significantly ameliorated the behavioral features, learning, and memory function (in Open field, Morris Water Maze, and Y-maze tests) in D-gal-induced aging in mice. These outcomes were correlated with a significant reduction of AGE levels and elevation of Cu, Zn-SOD, and CAT expressions and activities in the brain. UB inhibited the apoptosis of hippocampal neurons induced by D-gal, downregulated the JNK signaling pathway, prevented the cytochrome c release from isolated mitochondria, increased the activation of Akt and p44/42 MAPK, and promoted the neuronal survival via the PI3K/Akt pathways.	[145]
EA—50 mg/kg/day, intragastric (i.g.)	Mouse model of AD: Male APP/PS1 double-transgenic and wild-type (WT) C57BL/6 mice were divided into 4 groups: (1) WT, (2) WT+EA, (3) APP/PS1, (4) APP/PS1 + EA, received EA or the same volume of 10% DMSO for 60 days.	EA treatment improved learning and memory abilities and ameliorated cognitive deficits in APP/PS1 mice, reduced neuronal cell apoptosis, the expression of caspase-3 level and the amyloid aggregates in hippocampus. EA also significantly inhibited tau hyperphosphorylation and decreased the expression of pSer199-tau and pSer396-tau in the hippocampus of APP/PS1 mice. Moreover, EA treatment significantly increased the expression of pSer473-AKT and decreased the pTyr216-GSK3β levels in APP/PS1 mice.	[177]
EA—50 mg/kg, p.o.	AD animal model induced with AlCl_3_ in male Wistar rats divided into 4 groups: (1) control, (2) EA (50 mg/kg, p.o., for 4 weeks), (3) AD model (50 mg AlCl_3_/kg, p.o., for 4 weeks), (4) AD + EA (50 mg AlCl_3_/kg, p.o., for 4 weeks, followed by 50 mg EA/kg/day, p.o., for 2 weeks).	The discrimination index for the novel object recognition test (NORT) was significantly increased by EA therapy in AD rats. EA treatment significantly increased SOD, GSH, and TAC levels and decreased MDA levels in the serum of AD rats. The neurofibrillary tangles and neuritic plaques in the entorhinal cortex (ERC) sections were reduced in the AD+EA group. Antioxidant activity of EA treatment (increased SOD mRNA expression and modulated the amyloid precursor protein toxicity and caspase-3-mediated apoptosis) was correlated with the restoration of ERC thickness in AD+EA rats vs. AD rats.	[178]
EA—50 mg/kg bw/day (EA50) and 100 mg/kg bw/day (EA100), i.p., for 21 days	Animal model of memory impairment and anxiety induced by sleep deprivation (SD). C57BL/6J mice were divided into 4 groups: (1) control, (2) SD, (3) SD + EA50, (4) SD + EA100.	EA ameliorated learning and memory deficits and alleviated anxiety-like behaviors in SD mice. EA treatment improved neuron survival, reversed dendritic spine density, and reduced shrinkage and loss of neurons in the hippocampus of SD mice. EA restored the SOD and GPx activities, decreased MDA levels, and activated the Nrf2/HO-1 pathway in the hippocampus of SD mice. EA also reduced the IL-1β, IL-6, and TNF-α hippocampal levels, normalized the expression levels of TLR4, MyD88, NF-κB p65, and p-IκBα, and inhibited the TLR4-mediated innate immune responses. Moreover, EA showed neuroprotective effects on glutamate-induced toxicity via both the Nrf2 and TLR4 signaling pathways.	[179]
EA—50, 75, and 100 EA mg/kg, by gavages, 3 times daily for one week	Animal model of brain inflammation induced by cerebral ischemia/reperfusion (I/R). Male Wistar rats were divided into 6 groups: (1) control (surgery without any I/R) + vehicle (Veh); (2) I/R + Veh; (3–5) I/R + EA; (6) positive control (intact rats received 100 mg EA/kg).	Only the higher dose of EA (100 mg/kg) improved post-ischemic complications: it significantly increased the neurological signs scores, significantly reversed all tested behaviors, restored the BBB permeability, and decreased brain edema and the brain tissue cytokine levels (TNF-α and IL-1β) vs. I/R + Veh rats.	[180]
UA—300 mg, p.o.	Mouse model of Alzheimer’s disease (AD): APPswe/PS1ΔE9 (APP/PS1) mice received 300 mg of UA dissolved in 0.5% carboxymethylcellulose each day for 14 days vs. 2 control groups: APP/PS1 transgenic mice and wild-type mice that received only the vehicle (0.5% carboxymethylcellulose).	UA ameliorated spatial learning and memory impairment, prevented neuronal apoptosis in the cortex and hippocampus, and enhanced hippocampal neurogenesis in APP/PS1 mice. UA also decreased Aβ plaque deposit levels in the cortex and hippocampus, attenuated reactive gliosis, and significantly reduced microglia and astrocyte activation in APP/PS1 mice. UA treatment increased the expression of p-AMPK and decreased the activation of P65NF-κB and P38MAPK.	[181]
UA—2.5 mg/kg/day, i.p., for 8 weeks	A streptozotocin (STZ)- induced diabetic mouse model. Male CrljOri:CD1(ICR) mice were divided into 2 groups: (1) STZ-control and (2) STZ-UA.	UA treatment improved cognitive impairment, APP and BACE1 expressions, Tau phosphorylation, and Aβ deposition in STZ-induced diabetic mice. UA decreased blood glucose levels, but only in control mice and not in the STZ-injected mice.	[143]
UA—2.5 mg/kg bw; egpigallocatechin gallate (EGCG)—25 mg/kg bw	Animal model of late-onset AD using humanized homozygous amyloid beta knockin (hAbKI) mice were divided into 3 groups: (1) control, (2) UA (i.p., 3×/week for 4 months), (3) UA+EGCG (i.p., 3×/week for 4 months).	Both UA and UA + EGCG have been effective in counteracting the onset of pathological features of AD in hAbKI mice: enhanced phenotypic behavior, significantly increased mitochondrial biogenesis proteins (Nrf2, TFAM), both mitophagy (PINK1, Parkin) and synaptic proteins (synaptophysin, PSD95), as well as autophagy proteins (Beclin, ATG5, LC3B1, LC3B2, BCL2). Neuroinflammatory biomarkers (microglial marker Iba1 and astrocytic marker GFAP) were reduced, and the neuronal marker NeuN was significantly increased by both UA and UA + EGCG treatments. Dendritic spines and lengths, mitochondrial length, and mitophagosomal formations were also increased by both treatments. However, combined treatment UA+EGCG was stronger and more effective than only UA for most of the determinations performed.	[182]
*Juglans regia* (Gimcheon 1ho cultivar, GC)—lyophilized ethanolic extracts (obtained with 80% ethanol at 40 °C for 2 h), containing EA and ellagitannins (pedunculagin/casuariin isomer, strictinin, tellimagrandin I, EA-O-pentoside, and EA)—20 mg/kg bw (GC20) and 50 mg/kg bw (GC50), p.o.	A diabetic animal model with cognitive dysfunction in mice induced by a high-fat diet (HFD) for 12 weeks. Male C57BL/6 mice were divided into 4 groups: (1) control, (2) HFD, (3) HFD+GC20, (4) HFD + GC50. The lyophilized extracts were administered for 4 weeks.	GC significantly restored the behavioral and memory dysfunction in HFD-induced diabetic mice for both doses (GC 20 and GC50). GC also improved serum lipid profile and reduced white adipose tissue (WAT) and liver fat mass and showed antioxidant effects (reduced levels of MDA—in hepatic and cerebral tissues—and serum AGEs, and increased serum level of FRAP). GC inhibited AChE activity in cerebral tissue, suppressed AChE expression, and upregulated choline acetyltransferase expression vs. HFD group. GC restored mitochondrial membrane potential, regulated the mitochondrial ROS production and the protein expressions associated with synaptic disorders and neuronal apoptosis: decreased p-JNK, p-tau, and Aβ levels, upregulated p-Akt (Ser 473) and insulin-degrading enzyme (IDE), and downregulated BAX and caspase-3 expression levels vs. HFD group. Moreover, GC ameliorated TNF-α, IL-1β, p-NFκB, and caspase-1 expression levels and upregulated heme oxygenase-1 (HO-1) expression levels vs. HFD group.	[183]
Pomegranate juice (PJ) rich in ellagitannins (galloyl-hexoside, EA-hexoside, pedunculagin, casuarinin) and EA, containing 18.90 ± 0.96 g/L (EA equivalents per L of juice)	Rat model of Parkinson’s disease (PD) induced with rotenone (1.3 mg/kg bw/day, s.c., for 35 days). Male albino Wistar rats were divided into 4 groups: (1) control (vehicle only), (2) PJ (500 mg/kg bw/day, i.g.), (3) rotenone only (1.3 mg/kg bw/day, s.c.) from the 11th day (ROT), (4) PJ (500 mg/kg bw/day, i.g.) + rotenone from the 11th day (PJ + ROT). Experiment included 10 days pretreatment with PJ and 35 days with PJ + ROT combined treatment.	PJ improved postural stability in rats affected by rotenone, enhanced neuronal survival, protected against oxidative damage (reduced MDA levels and increased CAT, GPx, GST activities in midbrain) and α-synuclein aggregation, increased the activity of mitochondrial aldehyde dehydrogenase, and normalized the expression of anti-apoptotic Bcl-xL protein.	[184]
PJ	Rat model of Parkinson’s disease (PD) induced with rotenone (1.3 mg/kg bw/day, s.c., for 35 days). Male albino Wistar rats were divided into 4 groups: (1) control (vehicle only), (2) PJ (500 mg/kg bw/day, i.g.), (3) rotenone only (1.3 mg/kg bw/day, s.c.) from the 11th day (ROT), (4) PJ (500 mg/kg bw/day, i.g.) + rotenone from the 11th day (PJ + ROT). Experiment included 10 days pretreatment with PJ and 35 days with PJ + ROT combined treatment.	PJ treatment proved neuroprotection against PD: improved vertical activity in rotenone-injected rats mitigated the reduction of the olfactory discrimination index to the level observed in control animals, attenuated the depletion of the dopamine (DA) and 3,4-dihydroxy-phenylacetic acid (DOPAC) in the midbrain.	[185]
EA, α-lipoic acid (LA), myrtenal (Myrt)—50 mg/kg (i.p.)	Rat model of Parkinson’s disease (PD) induced with 6-hydroxydopamine (6-OHDA) (intrastriatal injection). Male Wistar rats divided into 5 groups: (1) control, (2) striatal 6-OHDA-lesioned control, (3–5) striatal 6-OHDA-lesioned rats pre-treated for 5 days with EA, LA, and Myrt.	All three compounds (EA, LA, and Myrt) improved learning and memory performance as well as neuromuscular coordination in rats with 6-OHDA-induced PD. Moreover, all these compounds significantly decreased lipid peroxidation (LPO) levels and restored catalase (CAT) activity and DA levels that were impaired by the challenge with 6-OHDA.	[186]
EA—10 mg/kg and 50 mg/kg, p.o.	Animal model of dopamine (DA) neuronal damage induced by lipopolysaccharides (LPS). Male Wistar rats were divided into 5 groups: (1) control, (2) EA (50 mg/kg), (3) LPS, (4) LPS+EA (10 mg/kg), and (5) LPS +EA (50 mg/kg).	EA (50 mg/kg) attenuated LPS-induced DA neuronal loss and ameliorated the decrease in tyrosine hydroxylase expression in the substantia nigra neurons. EA also attenuated the activation of the NLRP3 inflammasome in microglia and inhibited the NLRP3 inflammasome signaling pathway activated by LPS. The protein expressions of Iba-1 and proinflammatory cytokines IL-1β, TNF-α, and IL-18 were significantly suppressed by EA treatment.	[187]
EA—100 mg/kg and 200 mg/kg, i.p. and p.o.	Male albino Swiss mice—i.v. pentylenetetrazole (PTZ) seizure threshold test, maximal electroshock seizure test (MEST), grip-strength test, and chimney test, vs. valproic acid as reference drug.	EA (100 mg/kg) significantly increased the threshold of mice injected with PTZ for the first myoclonic twitch and generalized clonic seizures associated with the loss of the righting reflex, but not the threshold for forelimb tonus. In the MEST test, EA affected the threshold for the tonic hindlimb extension only at the high dose (200 mg/kg). EA did not have any effects on neuromuscular strength and motor coordination in mice at any of the doses tested.	[188]
Corilagin—10 mg/kg and 20 mg/kg, i.p.	Male Wistar rats—i.v. PTZ seizure threshold test.	Corilagin significantly reduced the epileptic events and improved cognitive function (in the Morris water maze (MWM) navigation test), reduced TNF-α and increased IL-10 levels, reduced ROS production, mitochondrial swelling, and carbonic anhydrase activity in the brain tissues in a dose-dependent manner. Corilagin treatment prevented structural damage of neurons and maintained the number of surviving cells vs. control group.	[189]
UA—10, 25, or 50 mg/kg/day, p.o.	Animal model of an experimental autoimmune encephalomyelitis (EAE) in C57BL/6 female mice immunized with MOG_35–55_.	The dose of 25 mg/kg significantly suppressed the progress of EAE. UA treatment decreased demyelination, significantly inhibited inflammatory cell infiltration, reduced neuroinflammation (lowered numbers of M1-type microglia and inhibited the activation of dendritic cells).	[190]
Pomegranate peel extract (PEm) rich in EA and punicalagin, vs. EA—50 mg/kg/day of EA	Animal model of EAE in C57BL/6 female mice immunized with MOG_35–55_.	Both EA and PEm showed comparable efficiencies to reduce the progression of the EAE and to ameliorate the clinical symptoms in mice. Spinal microgliosis and astrocytosis were significantly reduced by the PEm treatment as well as a clear reduction of the CD45 staining in the spinal cord was observed.	[191]
UA—2.5 mg/kg, i.p.	Mouse model of traumatic brain injury (TBI). Male C57BL/6J mice were divided into 3 groups: (1) control, (2) TBI + vehicle, (3) TBI + UA.	UA attenuated neuronal apoptosis following TBI (significantly reduced the cleaved caspase-3 expression and increased the Bcl-2 expression vs. TBI+vehicle), reinforced neuronal autophagy (increased the immunopositivity of LC3 and p62, two neuronal autophagy markers), and downregulated (PI3K)/Akt/mTOR (decreased Akt and mTOR expression levels) and Akt/IKK/NFκB signaling pathways (decreased the phosphorylation levels of IκB, IKKα, and NFκB).	[192]
Oenothein B—100 or 500 mg/kg/day, p.o.	Male ddY mice received oenothein B once a day for 3 days (from days 1 to 3) or 7 days (from days 1 to 7).	Oenothein B (only 100 mg/kg/day) activated extracellular signal-regulated kinase 2 (ERK2) in the hippocampal region of healthy mice, the ratio pERK2/ERK2 being significantly increased after 7 days of daily treatment. The activation of cAMP response element-binding protein (CREB), reflected by pCREB/CREB ratio, was slightly increased, but without statistical significance (*p* = 0.0866).	[193]
UA (30 μg UA/100 μL PBS, i.p.)	BALB/cJInv female mice were chronically infected with *Toxoplasma gondii* (*T. gondii*). UA treatment started 2 days post-infection and continued daily for 5 weeks vs. a control group (injected with vehicle).	All UA-injected mice survived throughout the entire experiment vs. 60% in the control group (40% of the infected control mice died 10-days post-infection). UA treatment inhibited cyst formation in the brain and altered the response of infected mice toward cat odor.	[194]

AChE—acetylcholinesterase; AD—Alzheimer’s disease; D-gal—D-galactose; EA—ellagic acid; EAE—experimental autoimmune encephalomyelitis; EGCG—egpigallocatechin gallate; GC—*Juglans regia*, Gimcheon 1ho cultivar; HFD—high-fat diet; Iba-1—ionized calcium-binding adapter molecule-1; i.g.—intragastric; IL—interleukin; i.p.—intraperiotoneal; i.v.—intravenous; MEST—maximal electroshock seizure test; MOG_35–55_—myelin oligodendrocyte protein 35–55 peptide; NLRP3—Nod-like receptor protein 3; 6-OHDA—6-hydroxydopamine; PBS—phosphate-buffered saline; PD—Parkinson’s disease; PEm—pomegranate peel extract; PJ—pomegranate juice; p.o.—per os; PTZ—pentylenetetrazole; ROT—rotenone; SD—sleep deprivation; TNF-α—tumor necrosis factor-α; UA—urolithin A; UB—urolithin B; WT—wild-type.

## Data Availability

Data are contained within the article.

## Abbreviations

ABTS—2,2′-azinobis-(3-ethylbenzothiazoline-6-sulfonic acid; AchE—acetylcholinesterase; AD—Alzheimer’s disease; AhR—aryl hydrocarbon receptor; Akt—protein kinase B; AMPK—AMP-activated protein kinase; APP—amyloid precursor protein; ARE—antioxidant response elements; BACE1—β-secretase-1; BBB—blood-brain barrier; bw—body weight; BDNF—brain-derived neurotrophic factor; CAT—catalase; CCA—cholangiocarcinoma; CNS—central nervous system; COX—cyclooxygenase; CRC—colorectal cancer; CREB—cAMP response element-binding protein; CRP—C-reactive protein; DA—dopamine; DE—dimeric ellagitannins; D-gal—D-galactose; DPPH—2,2-diphenyl-1-picrylhydrazyl; EA—ellagic acid; EAE—experimental autoimmune encephalomyelitis; EGCG—epigallocatechin gallate; EPM—elevated plus maze; ERC—entorhinal cortex; ERK—extracellular signal-regulated kinases; ETs—Ellagitannins; FRAP—ferric reducing ability of plasma; GC—*Juglans regia*, Gimcheon 1ho cultivar; GPx—glutathione peroxidase; GSH—total glutathione; (GSK)3β—glycogen synthase kinase; GSSG—oxidized glutathione; HAT—hydrogen atom transfer; HBV—hepatitis B virus; HCC—hepatocellular carcinoma; HDL—high-density lipoprotein; HFD—high-fat diet; HHDP—hexahydroxydiphenic acid; HO-1—heme oxygenase-1; Iba-1—ionized calcium-binding adapter molecule-1; IBS—irritable bowel syndrome; i.g.—intragastric; IκB—inhibitor of kappa B; IL—Interleukin; iNOS—induced NO synthase; i.p.—intraperitoneal; IPA—indole propionate; i.v.—intravenous; JNK—Jun N-terminus kinase; LDL—low-density lipoprotein; LOX—lipoxygenase; LPS—lipopolysaccharides; MAO—monoamine oxidase; MAPK—mitogen-activated protein kinase; MDA—malondialdehyde; ME—monomeric ellagitannins; MEST—maximal electroshock seizure test; MMP—matrix metalloproteinase; MS—multiple sclerosis; mTOR—mammalian target of rapamycin; mtROS—mitochondrial ROS; MWM—Morris water maze; NF-кB—nuclear factor kappa B; NLRP3—Nod-like receptor protein 3; NORT—novel object recognition test; Nrf2—nuclear factor erythroid 2-related factor 2; OF—open field; 6-OHDA—6-hydroxydopamine; ORAC—oxygen radical absorbance capacity; OXPHOS—oxidative phosphorylation; PD—Parkinson’s disease; PDAC—pancreatic ductal adenocarcinoma; PEm—pomegranate peel extract; PGC-1α—peroxisome proliferator-activated receptor gamma coactivator; PI3K—phosphatidylinositol 3-kinase; PID—pelvic inflammatory disease; PJ—pomegranate juice; p.o.—per os; PPAR—peroxisome proliferator-activated receptor; PTZ—pentylenetetrazole; QOL—quality of life; RCT—randomized clinical trial; ROS—reactive oxygen species; ROT—rotenone; SD—sleep disorder; SET—single electron transfer; SIRT—sirtuins—silent information regulators; SNpc—substantia nigra pars compacta; SOD—superoxide dismutase; STZ—streptozotocin; TC—total cholesterol; TEAC—trolox equivalent antioxidant capacity; TG—triglycerides; TGM2—transglutaminase type 2; TLR4—Toll-like receptor 4; TNF—tumor necrosis factor; Trp—tryptophan; U—urolithin; WT—wild-type.
